# Population genomics of seal lice provides insights into the postglacial history of northern European seals

**DOI:** 10.1111/mec.17523

**Published:** 2024-09-09

**Authors:** Ludmila Sromek, Kevin P. Johnson, Mervi Kunnasranta, Eeva Ylinen, Stephany Virrueta Herrera, Elena Andrievskaya, Vyacheslav Alexeev, Olga Rusinek, Aqqalu Rosing‐Asvid, Tommi Nyman

**Affiliations:** ^1^ Department of Marine Ecosystems Functioning Institute of Oceanography, University of Gdansk Gdynia Poland; ^2^ Illinois Natural History Survey Prairie Research Institute, University of Illinois Champaign Illinois USA; ^3^ Department of Environmental and Biological Sciences University of Eastern Finland Joensuu Finland; ^4^ Natural Resources Institute Finland Joensuu Finland; ^5^ School of Environmental Sustainability, Loyola University Chicago Chicago Illinois USA; ^6^ The Baltic Ringed Seal Foundation St. Petersburg Russia; ^7^ Baikal Museum of the Siberian Branch of the Russian Academy of Sciences Listvyanka Russia; ^8^ Greenland Institute of Natural Resources Nuuk Greenland; ^9^ Department of Ecosystems in the Barents Region, Svanhovd Research Station Norwegian Institute of Bioeconomy Research Svanvik Norway

**Keywords:** coalescent simulations, demographic history, genetic diversity, host‐associated genetic differentiation, host–parasite interactions, phylogeography

## Abstract

Genetic analyses of host‐specific parasites can elucidate the evolutionary histories and biological features of their hosts. Here, we used population‐genomic analyses of ectoparasitic seal lice (*Echinophthirius horridus*) to shed light on the postglacial history of seals in the Arctic Ocean and the Baltic Sea region. One key question was the enigmatic origin of relict landlocked ringed seal populations in lakes Saimaa and Ladoga in northern Europe. We found that that lice of four postglacially diverged subspecies of the ringed seal (*Pusa hispida*) and Baltic gray seal (*Halichoerus grypus*), like their hosts, form genetically differentiated entities. Using coalescent‐based demographic inference, we show that the sequence of divergences of the louse populations is consistent with the geological history of lake formation. In addition, local effective population sizes of the lice are generally proportional to the census sizes of their respective seal host populations. Genome‐based reconstructions of long‐term effective population sizes revealed clear differences among louse populations associated with gray versus ringed seals, with apparent links to Pleistocene and Holocene climatic variation as well as to the isolation histories of ringed seal subspecies. Interestingly, our analyses also revealed ancient gene flow between the lice of Baltic gray and ringed seals, suggesting that the distributions of Baltic seals overlapped to a greater extent in the past than is the case today. Taken together, our results demonstrate how genomic information from specialized parasites with higher mutation and substitution rates than their hosts can potentially illuminate finer scale population genetic patterns than similar data from their hosts.

## 1 INTRODUCTION

Parasites can provide key additional information on the ecology, biology and evolutionary history of their hosts. For example, genetic analyses of parasites have been used to gain insights into past foraging habits and food sources of hominids (Hoberg et al., 2001), the origin of clothing (Kittler et al., 2003), and contacts between modern and archaic humans (Reed et al., 2004). Within the field of phylogeography, parasites have provided evidence about past dispersal events, Quaternary refuges, and colonization routes (Nieberding et al., 2004; Whiteman et al., 2007; Wickström et al., 2003). Beyond the study of such historical processes, parasites have been widely used as biological markers to discriminate between extant stocks, migration routes, and nursery grounds (Catalano et al., 2014; Gagne et al., 2022; Mattiucci et al., 2015; Moore et al., 2019). Parasite genetic data can also provide information about host population connectivity (Sromek et al., 2023; Virrueta Herrera et al., 2022) and can detect recent changes in dispersal patterns that may be difficult to assess using host genetics alone (Gagne et al., 2022; Speer et al., 2019). An important practical advantage for biodiversity conservation is that many parasites have considerably smaller genomes (Coghlan et al., 2019) than their vertebrate hosts (Kapusta et al., 2017), which makes genome‐level analyses faster and more cost efficient (Johnson, 2019; Virrueta Herrera et al., 2022).

For a parasite to be useful as a marker of its host's evolutionary history, it must share a common history of isolation and diversification with the host (Nieberding & Olivieri, 2007). This condition is most often met by host‐specific directly transmitted permanent parasites that do not have a free‐living phase (Gagne et al., 2022; Geraerts et al., 2022; Nieberding & Olivieri, 2007; Whiteman et al., 2007). Such parasites typically undergo more generations per unit time than their hosts (Johnson et al., 2014; Light & Hafner, 2007; Whiteman & Parker, 2005), which may be associated with higher rates of mutation and genetic substitution. At the same time, within‐population genetic variation may be lowered by inbreeding caused by infrapopulation structure (Criscione & Blouin, 2005; Doña & Johnson, 2023) and successive founder events during colonization of new host individuals (Papkou et al., 2016). These properties are particularly beneficial for reconstructing recent divergence events in long‐lived hosts with large subpopulations (Gagne et al., 2022). In such cases, the level of incomplete lineage sorting may be lower between parasite populations, and the parasite tree may therefore better reflect the shared history of the parasite and its hosts than the host tree (Nieberding & Olivieri, 2007). This ‘magnifying glass effect’ (Nieberding & Morand, 2006) makes parasites a particularly compelling system for studies in population genetics.

**FIGURE 1:**
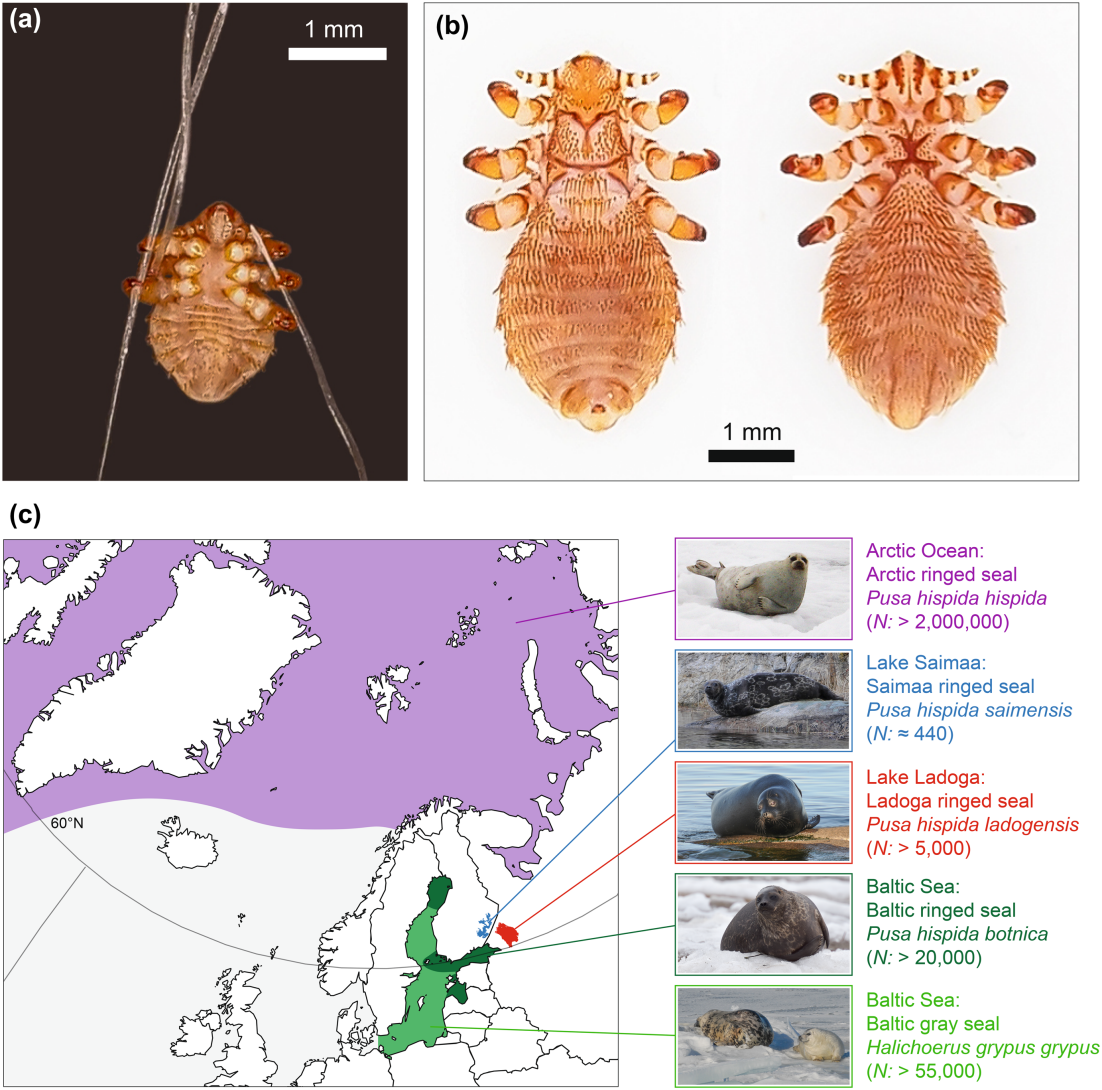
(a) Nymph of the seal louse *Echinophthirius horridus* clinging to hairs of a Saimaa ringed seal pup. (b) Dorsal and ventral view of an adult male *E. horridus* from Baikal seal. (c) Geographical distributions and estimated current population sizes of the focal northern European seal populations from which *E. horridus* lice were collected for the present study.

**FIGURE 2:**
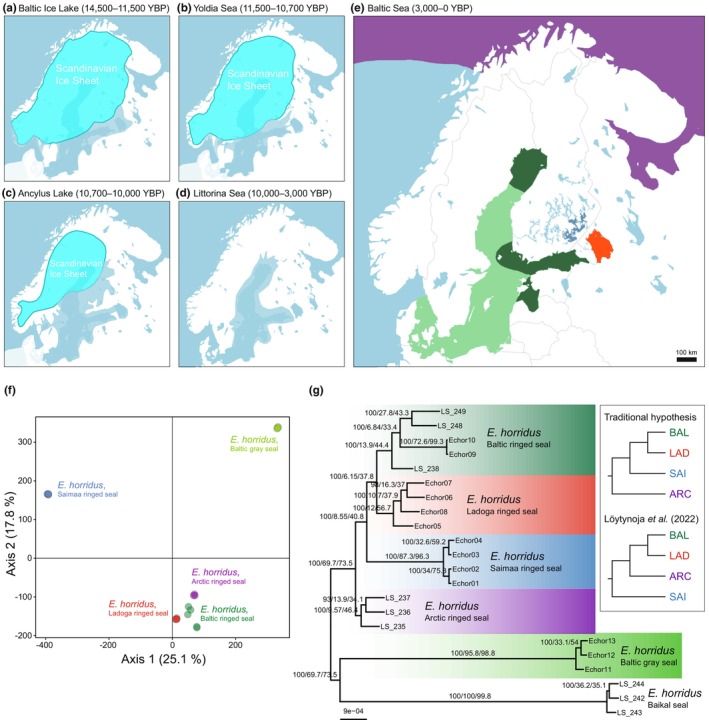
(a–e) The main periods of the Baltic Sea basin and (e) the current Baltic Sea during and after the disappearance of the Scandinavian Ice Sheet. In (e), the distributions of the focal seal host populations from which *Echinophthirius horridus* lice were collected for the present study are indicated with the same colours as in Figure 1c. (f) Principal component analysis (PCA) plot illustrating genetic relatedness among seal louse individuals based on the LD‐pruned SNP dataset. The first two principal components are shown, and dots are coloured according to the seal host populations. (g) Concatenated ML tree for 22 *E. horridus* individuals inferred based on the nuclear phylogenomic dataset. Branch lengths are proportional to the number of substitutions per site, and numbers above branches separated by slashes are: Ultrafast bootstrap support value, gene concordance factor (gCF) and site concordance factor (sCF), respectively, for that branch (for discordance factors, see Table S3). The inset in (g) shows alternative topologies corresponding to the traditional hypothesis of the origin of ringed seal subspecies in northern Europe (Davies, 1958; Ukkonen, 2002) and the phylogenetic hypothesis proposed by Löytynoja et al. (2022) based on data on seal genomes; populations are abbreviated and coloured as in Figure 3. Maps in panels a–e were drawn based on templates provided by Datawrapper GmbH.

Here, we applied detailed population‐genomic analyses of the seal louse *Echinophthirius horridus* (Psocodea: Echinophthiriidae) to elucidate the postglacial history of ringed seal (*Pusa hispida*) subspecies inhabiting the Arctic Ocean, the Baltic Sea, and the large lakes Saimaa and Ladoga in northern Europe (Figure 1). The Baltic ringed seal (*P. h. botnica*), the Saimaa ringed seal (*P. h. saimensis*), and the Ladoga ringed seal (*P. h. ladogensis*) are believed to descend from Arctic ringed seals (*P. h. hispida*) that colonized the Baltic Sea basin after the retreat of the Scandinavian Ice Sheet some 10,200–10,900 years ago (Schmölcke, 2008; Ukkonen, 2002; Ukkonen et al., 2014). Due to progressive land uplift resulting from the disappearance of the thick continental ice sheet, parts of this ancestral Baltic population were subsequently trapped in the emerging lakes Saimaa and Ladoga. Based on the geological history of the Baltic Sea region (Figure 2a–e), the endemic seal population of Lake Saimaa presumedly has been isolated for around 9500 years (Ukkonen et al., 2014). By contrast, the effective isolation of the relict subspecies inhabiting Lake Ladoga is probably shorter, as the Ladoga basin may have been broadly connected with the Baltic Sea until around 4000 years ago (Kuznetsov et al., 2022; Saarnisto, 2011). Despite their relatively short evolutionary histories, the Baltic and landlocked ringed seal subspecies are genetically, morphologically and behaviourally differentiated from each other as well as from the Arctic ringed seal (Kunnasranta et al., 2021).

While the well‐known geological history of northern Europe predicts a clear time frame and sequence of events for the colonization of the Baltic Sea and the two postglacial lakes by ringed seals (Figure 2a–e), recent genetic studies on Saimaa ringed seals have challenged the concordance between geology and genetics. A study of mitochondrial control‐region sequences in museum and extant Saimaa ringed seal samples by Heino et al. (2023) did not confirm its close affinity with the Baltic ringed seal population, but rather suggested links with North American ringed seals. Similarly, a phylogenetic tree based on whole‐genome resequencing data by Löytynoja et al. (2022) placed the Saimaa ringed seal at a basal position, as sister to a clade formed by Arctic, Baltic and Ladoga seals. Although many regional trees were incongruent with the consensus topology (Löytynoja et al., 2022), the presence of many unique SNPs in Saimaa ringed seals also suggests a more ancient origin of the subspecies (Löytynoja et al., 2023). Resolving the relationships among northern European ringed seal populations is evidently complicated not only by the relatively rapid and sequential divergence of the subspecies, but also by the extremely large size of the Arctic ringed seal population, which consists of several million individuals in a largely panmictic, circumpolar population (Fulton & Strobeck, 2010; Lang et al., 2021; Martinez‐Bakker et al., 2013). Under a simple allopatric speciation model, reciprocal monophyly at more than 50% of loci is attained after about 4–5 *N*_e_ (effective population size) generations of population isolation (Hudson & Coyne, 2002). For the ringed seal, with a generation time of c. 11 years and an effective population size in the tens of thousands (Löytynoja et al., 2023; Palo et al., 2001; Peart et al., 2020), reaching this level would take millions of years.

In this context, the seal louse *Echinophthirius horridus* emerges as a promising ‘independent marker’ of seal evolution and demography due to its reduced generation time, which is in the range of a few months rather than years (Aznar et al., 2009; Thompson et al., 1998), and therefore an order of magnitude shorter than that of its seal hosts. The demographic and population history of *E. horridus* is expected to be tightly linked to that of its hosts, because seal lice are permanent, obligate ectoparasites whose transmission requires close physical contact between host individuals (Leidenberger et al., 2007; Leonardi et al., 2013, 2021). A phylogenomic analysis of other lice from southern‐hemisphere seals and sea lions by Leonardi et al. (2019) indeed showed a strong pattern of codivergence, with a low degree of switching among host species. At the population level, a recent survey of *E. horridus* from Lake Saimaa revealed that spatial genetic differentiation in the louse population (Virrueta Herrera et al., 2022) closely matches the spatial structuring present within the lake‐endemic Saimaa ringed seal population (Löytynoja et al., 2023; Valtonen et al., 2012, 2014).

In this study, we integrated population‐ and phylogenomic analyses with demographic modelling in a coalescent framework to reconstruct the evolutionary history of *Echinophthirius* lice parasitizing ringed seal subspecies that have diverged after the Pleistocene in northern Europe. Our underlying assumption was that genome‐wide analyses of these specialized, rapidly evolving ectoparasites would be useful for elucidating the puzzling history of northern European seals because, temporally, the colonization of the Baltic Sea basin and large postglacial lakes by ringed seals spans a window between the species‐level divergences studied by Leonardi et al. (2019) and the fine‐scale population structuring investigated by Virrueta Herrera et al. (2022). As the first step, we conducted whole‐genome sequencing of *E. horridus* specimens collected from Arctic, Baltic, Ladoga, and Saimaa ringed seals, as well as the partially sympatric Baltic gray seal. We then used the resultant nuclear and mitochondrial datasets to estimate levels of population differentiation and genetic diversity, and to infer the sequence and time frame of divergence events among the focal populations through phylogenomic analyses and coalescent simulations. Finally, we estimated population‐size trajectories through the Pleistocene based on individual genome assemblies. Specifically, we hypothesized that: (1) *E. horridus* populations on different seal host species and subspecies should be genetically differentiated; (2) genetic diversity within each seal louse population should reflect the population size and genetic diversity of their host (sub)species; (3) the order and timing of divergences among seal louse populations should reflect the sequential emergence of the Baltic Sea basin and lakes Saimaa and Ladoga during the gradual disappearance of the Scandinavian Ice Sheet and (4) long‐term population trajectories should show signatures of past climatic variation. Additionally, because the distributions of Baltic ringed and gray seals overlap partially, we used multiple different statistical approaches to test for the presence of hybridization between the focal louse populations.

## 2 METHODS

### 2.1 Sample collection

We sampled a total of 22 *E. horridus* seal lice for our analyses. To avoid sampling closely related individuals (Virrueta Herrera et al., 2022), we aimed as far as possible to sample louse specimens from different seal individuals within each focal population. Hence, we sampled one louse each from four Saimaa ringed seals, one louse each from four Ladoga ringed seals, a total of five lice from three Baltic ringed seals, three lice from one Arctic ringed seal and one louse each from three Baltic gray seals (Table S1). In addition to these 19 lice from the focal northern European seal populations, we sequenced one specimen each from three Baikal seals (*Pusa sibirica*) to serve as an outgroup during phylogeny reconstruction. Lice were collected opportunistically through 2009–2020 from pups handled during radio telemetry studies (Saimaa and Baltic ringed seals; permit numbers ESAELY/433/07.01/2012, ESA‐2008‐L‐519‐254, ESAVI/8269/04.10.07/2013, and ESAVI‐2010‐08380/Ym‐23), during necropsies of stranded and by‐caught seals (Saimaa and Ladoga ringed seals and Baltic gray seals; permits MMM 234/400/2008 and VARELY/3480/2016), from a seal skin sold to a tannery (Arctic ringed seal), and from seals hunted during the regular hunting season (Baltic ringed seals and Baikal seals). Lice were preserved in 99.5% ethanol or RNAlater and stored at −20°C.

### 2.2 DNA extraction and whole‐genome sequencing

DNA was isolated using the QIAmp DNA Micro Kit (Qiagen) according to the manufacturer's protocol with the following modifications: incubation time was increased from 1–3 to 24–48 h and buffer AE was replaced with buffer EB. DNA extracts were quantified with a Qubit fluorometer (Thermo Fisher). Individual‐specific whole‐genome sequencing libraries were prepared using either KAPA Hyper Prep Kit (Kapa Biosystems) or NEBNext Ultra II DNA Library Prep Kit (New England Biolabs). Multiplexed libraries were then sequenced on NovaSeq 6000 platform using 150‐bp paired‐end mode.

### 2.3 Genome assembly

To obtain a reference for read mapping and genotyping, we first assembled the nuclear genome of *E. horridus*. For this, we followed the analysis pipeline described by Zhang et al. (2019). Raw sequences were firstly compressed into clumps, deduplicated, quality trimmed and normalized using BBTools suite v. 37.62 (Bushnell, 2014), and then error corrected using Lighter v. 1.1.2 (Song et al., 2014). Next, contig assembly was performed using Minia v. 3.2.1 (Chikhi & Rizk, 2013) and redundant contigs were removed using Redundans v. 0.14a (Pryszcz & Gabaldón, 2016). Finally, scaffolding and gap‐filling were performed with BESST v. 2.2.8 (Sahlin et al., 2014) and GapCloser v. 1.12 (Luo et al., 2012), respectively. Assembly completeness was assessed with BUSCO v. 5.4.5 (Manni et al., 2021) against the insect reference gene set (*n* = 1367). The genomes of several individuals were assembled using this fast pipeline, and the genome of the individual (Echor03 from Lake Saimaa) with highest completeness was used for further analyses. To detect potential contaminants, assembled scaffolds were blasted against the NCBI nucleotide database, using BLAST 2.10.0+ (Zhang et al., 2000) with an ‘*E*‐value’ significance threshold of 1 × 10^−6^. A total of 91 scaffolds that had hits to seals, otter or bacterial sequences at a length of at least 200 bp were discarded from further analysis. Six scaffolds that contained louse mitochondrial genes were also excluded.

### 2.4 Nuclear datasets

For use in the different phylogenomic and population‐genomic analyses below, we created three different nuclear datasets (Sromek et al., 2024), which were then modified as needed for each specific analysis (Figure S1). In the first step, the adapter‐and‐quality‐trimmed reads were mapped to our draft *E. horridus* genome using the local alignment mode of Bowtie2 v. 2.4.4 (Langmead & Salzberg, 2012). Duplicate reads were not filtered because quality checks of raw sequence data in FastQC (Andrews, 2010) did not indicate any clear issues with the degree of sequence duplication. Because per‐sample coverages are high in relation to the estimated levels of duplication (Table S1), the inclusion of duplicate reads is not expected to influence genotype calls (Rochette et al., 2023). Variant calling was performed using the ‘HaplotypeCaller’ function in GATK v. 3.8 (Van der Auwera & O'Connor, 2020) for each sample separately. Samples from the same host population were then jointly genotyped using the ‘GenotypeGVCF’ function in GATK, retrieving both variant and non‐variant sites. Samples from different populations were then merged into one VCF file using BCFtools (Danecek et al., 2021).

For analyses of host‐associated genetic differentiation, genetic diversity, hybridization and demographic history, we extracted biallelic SNPs from the GATK file using BCFtools and filtered them with the following parameters: site Phred quality score >30, a maximum site depth of 3067x (twice the average site read depth), a minimum genotype depth of 10x and sample‐level genotype quality >30. SNPs heterozygous in more than 14 individuals were also removed as potential mapping errors in repetitive or structural variants. We did not mask repeats, as by filtering for unusually high site read depth, genotyping quality, and an excess of heterozygotes we should have excluded most variants in these regions, and because louse genomes generally seem to have a low fraction of repeats (Xu et al., 2024). Finally, SNPs with missing genotypes for any individual were excluded using VCFtools v. 0.1.17 (Danecek et al., 2011), resulting in dataset below referred to as the filtered SNP dataset. An additional dataset controlling for linkage was obtained by pruning the filtered SNP dataset for *r* ^2^ < .1 in PLINK v.1.90 (Chang et al., 2015) considering 50 SNP windows and moving 10 SNPs per set (‐indep‐pairwise 50 10 0.1). This dataset is below referred to as the LD‐pruned SNP dataset.

To obtain a dataset for reconstructing phylogenetic relationships and examining genealogical discordance across the genome, we split our assembled scaffolds into 50‐kb non‐overlapping windows. The VCF file from GATK was split into windows using BCFtools and converted to PHYLIP format with the vcf2phylip script (Ortiz, 2019). Scaffolds shorter than 50 kb and nucleotide positions present in less than four individuals were discarded. This dataset consisting of 50‐kb genomic windows is below referred to as the phylogenomic dataset.

### 2.5 Mitochondrial dataset

In addition to the nuclear datasets, we used the sequencing outputs to assemble the sequences of seven maternally inherited mitochondrial coding genes. For this, we used aTRAM v. 2.3.0 (Allen et al., 2018), with mitochondrial amino acid sequences from the human louse, *Pediculus humanus* (Shao et al., 2009) as the reference target. We used this approach because louse mitochondrial genomes are frequently fragmented (Shao et al., 2009; Sweet et al., 2022) and because including only coding sequences increases the reliability of alignments. To make aTRAM libraries, we subsampled 8.5 million quality‐trimmed reads from each individual using BBTools suite. Assemblies were performed using the Velvet assembler and default aTRAM parameters. Of the 13 mitochondrial genes, seven (i.e. COI, COII, COIII, CYTB, ND1, ND4 and ND5) were successfully assembled in all individuals. Since for some individuals the final assembled sequences were not complete or contained putative bacterial contaminants, the most complete assembled sequences were used as targets for read mapping in Bowtie2. Consensus sequences were then constructed from the mapped reads using SAMtools and BCFtools (Li, 2011), and converted to fasta files using scripts written by Andrew D. Sweet (https://github.com/adsweet/louse_genomes). Individual mitochondrial genes were aligned in MAFFT v. 7.505 (Katoh & Standley, 2013) using the accurate ‐linsi option.

### 2.6 Inference of host‐associated genetic divergence and genetic diversity

To visualize overall relationships among louse individuals collected from different hosts, we used principal component analysis (PCA). PCA was computed based on the LD‐pruned SNP dataset using the *dudi.pca* function in ade4 v.1.7.20 (Dray & Dufour, 2007) of R v. 4.2.2 (R Core Team, 2022). Next, we estimated SNP statistics (i.e. the number of polymorphic and fixed SNPs in each population) from the filtered SNP dataset using BCFtools, and used Venn diagrams drawn with the R package ggVennDiagram (Gao, 2021) to examine patterns of SNP sharing between louse populations from different hosts. Because the population‐level number of polymorphic sites may be influenced by sample size, we also calculated this index based on all possible subsamples of three individuals for lice from Baltic, Ladoga and Saimaa ringed seals. Genetic diversity within each sample was also evaluated based on per‐individual observed heterozygosity calculated from the LD‐pruned SNP dataset and an intermediate dataset of filtered genotypes (i.e. including also invariant sites and before LD pruning; Figure S1).

For the mitochondrial data, we estimated percent pairwise sequence divergences (uncorrected p‐distances) between and within populations using the R package APE v. 5.7.1 (Paradis & Schliep, 2019). These calculations were based solely on sequences (1428 bp) of the COI standard ‘DNA barcoding gene’ (Hebert et al., 2003), which is extensively used for species identification and delimitation in insects (Lee et al., 2022; Wilson et al., 2017).

### 2.7 Phylogeny estimation

We estimated maximum likelihood (ML) phylogenetic trees for the concatenated nuclear sequence dataset and each 50‐kb window in IQ‐TREE v. 2.2.2.7 (Minh, Schmidt, et al., 2020). The concatenated species tree was inferred using the edge‐linked partition model (Chernomor et al., 2016), with model selection (Kalyaanamoorthy et al., 2017) performed separately for each partition (genomic window). Branch support was inferred based on 1000 ultrafast bootstrap replicates (Hoang et al., 2018). Gene trees estimated for individual genomic windows were used to calculate the gene concordance factor (gCF) for each branch of the species tree, that is, the percentage of decisive gene trees supporting that particular branch (Minh, Hahn, & Lanfear, 2020). The site concordance factor (sCF), defined as the fraction of decisive alignment sites supporting each particular branch, was calculated using 100 randomly sampled quartets. The root position was inferred using RootDigger v. 1.7.0 (Bettisworth & Stamatakis, 2021) with default parameters.

IQ‐TREE was also used for estimating an ML tree for the seven‐gene mitochondrial sequence alignment. The analysis was partitioned according to gene and codon positions (1+2 vs. 3), with model selection for each partition. Branch support was estimated using 1000 ultrafast bootstrap replicates.

### 2.8 Estimation of hybridization

To further explore ancestral relationships between louse populations, we performed TreeMix v. 1.13 (Pickrell & Pritchard, 2012) analysis, which uses allele frequency data for inferring past population splits and admixture. This analysis involves adding migration edges to the population tree and evaluating whether they reduce deviations in the residual covariance matrix and improve the model fit. The TreeMix input file was created from the LD‐pruned SNP dataset using PPP Input File Generator (Webb et al., 2021). Up to five migration events were fitted on the tree, with five independent runs performed for each scenario. Lice from Lake Baikal were set as the outgroup.

Since the patterns of phylogenetic discordance and the results of the TreeMix analyses were consistent with admixture between lice from Baltic ringed and gray seals (see below), we tested this possibility using *D* statistics (ABBA‐BABA tests) (Durand et al., 2011; Green et al., 2010). In the analyses, lice from Baikal seal were used as the outgroup [O], lice from Baltic gray seal as a potential donor species (P3), and lice from Baltic, Saimaa and Ladoga ringed seals as potential recipient populations (P2). Lice from Ladoga, Saimaa and Arctic ringed seals were successively used as P1, according to the method described by Martin et al. (2013). This allowed us to estimate admixture between lice from Baltic ringed and gray seals across three different time periods: a recent period, subsequent to the divergence of lice from Baltic and Ladoga ringed seals; an intermediate period, subsequent to the divergence of lice from Baltic and Saimaa ringed seals; and the longest period, subsequent to the divergence of lice from the Baltic and Arctic ringed seals. The relative abundance of ABBA and BABA patterns was compared using the *D* statistic, based on derived allele frequencies at each SNP in the filtered SNP dataset. Sites that were not homozygous for the same allele in the three outgroup lice from Baikal seal were excluded, resulting in dataset of 3,484,553 SNPs. The admixture proportion *f* was calculated by comparing the observed excess of ABBA over BABA sites with that expected under complete admixture. To approximate the expectation under complete admixture, we split the P3 population into two (P3a and P3b) and counted ABBA and BABA sites using P1, P3a and P3b. A 1‐Mb block jack‐knifing procedure was then used to calculate the mean and variance of both the *D* statistic and *f* value using R scripts by Martin et al. (2013).

### 2.9 Inference of demographic history

We used coalescent simulations in fastsimcoal2 (Excoffier et al., 2021) and analyses based on the Pairwise Sequentially Markovian Coalescent (PSMC) model (Li & Durbin, 2011) to infer the demographic histories of the focal northern European seal louse populations. The aim of the coalescent simulations was to evaluate how different hypothetical models of the origin of lake seal louse populations fit our data, and we used the PSMC analyses to infer population‐size trajectories over longer time periods.

The coalescent simulations in fastsimcoal2 were based on multidimensional folded site frequency spectra (SFSs) built from the filtered SNP dataset using R scripts by Vitor Sousa (http://cmpg.unibe.ch/software/fastsimcoal2/additionalScripts.html). One SNP per 1000 bp block was sampled for model selection to reduce the effect of non‐independence of markers, and all SNPs were used for estimation of parameters. For each demographic model, 30 independent fastsimcoal runs were conducted, with each run consisting of 40 expectation‐conditional maximization cycles rounds and 100,000 coalescent simulations. The run yielding the highest ML value for each model was then selected for calculating Akaike's Information Criterion (AIC).

We initially formulated and tested six different five‐population models (Figure S2). The first three models corresponded to the three possible topologies between the lice from Baltic and landlocked ringed seal populations. The fourth model tested the possibility that the divergence of lice of Saimaa ringed seal pre‐dates the divergence of the common ancestor of lice from Baltic and Ladoga ringed seals from lice of Arctic ringed seals (as suggested by the genetic uniqueness of the Saimaa ringed seal). The fifth model tested the possibility that lice from both Saimaa and Ladoga descend from an unsampled ‘ghost’ lineage and are not closely related to lice from the Baltic ringed seal. Based on the results of the TreeMix analyses and ABBA‐BABA tests (see below), the sixth model included an admixture event between lice from Baltic gray and ringed seals. Of these six models, the model with admixture had the highest likelihood, so we added two more models with continuous and ancient gene flow between lice of Baltic gray and ringed seals (Figure S2). Details of each tested model (template and parameter estimation files with all defined parameters and their search ranges) are available in the file package deposited on Zenodo (see Data availability statement).

We then performed final parameter estimates under the best supported model using all SNPs and non‐parametric bootstrapping. Bootstrap replicates (*n* = 100), used to calculate 95% confidence intervals of the estimates, were obtained by resampling with replacement blocks of 1000 bp. To convert the inferred parameters into demographic units, we used a mutation rate of 3.5 × 10^−9^ per site and generation, based on an estimate from *Drosophila melanogaster* (Keightley et al., 2009). The generation time of *E. horridus* is likewise unknown (Herzog, Siebert, & Lehnert, 2024; Herzog, Wohlsein, et al., 2024). Different species of seal lice take from 18 to 26 days to complete their life cycle, but the lice can reproduce only when their hosts are hauled out on land or ice during their reproductive or moulting seasons (Aznar et al., 2009; Kim, 1975; Murray et al., 1965). Depending on the biology of the host (Soto et al., 2024) this would mean two to three generations per year (Aznar et al., 2009; Kim, 2006). Because the duration of the life cycle of *E. horridus* was suggested to be longer than those of Antarctic lice (Thompson et al., 1998), we conservatively assumed two generations per year.

The PSMC method uses the genome sequence of a single individual to identify how the coalescent rate varies across the genome, and from these values estimates changes in effective population size over time (Li & Durbin, 2011). To prepare the input for the PSMC analyses, we obtained a consensus genome sequence from the mapped BAM files for each ingroup louse individual using the ‘mpileup’ and ‘call’ commands in BCFtools. Several filters were added to keep only those consensus sequences with high confidence: (1) the minimum mapping quality for an alignment (‐q) was set to 5 and minimum base quality (‐Q) to 28, (2) sites with sequencing depths smaller (‐d) than 10 and larger (‐D) than twice of the average depth of the aligned genome were excluded and (3) consensus sequences shorter than 10 kb and those with consensus quality lower than 20 were filtered out. The first two filters were applied in BCFtools and the third one in the PSMC package. For PSMC analyses, we tested different settings for atomic time intervals (‐p) and the upper limit for the most recent common ancestor (‐t) to select those that maximized the number of time intervals with at least 20 recombination events. The final setting was ‘‐p 45*2 ‐t10’. The reconstructed population history was plotted using the plotPsmc.r script from the study by Liu and Hansen (2017). The mutation rate and the generation time were assumed as in the above fastsimcoal2 analyses (*μ* = 3.5 × 10^−9^, *g* = 0.5).

## 3 RESULTS

### 3.1 Draft genome assembly and datasets

The sequencing runs produced between 41 and 257 million reads per sample (Table S1). Our final draft genome assembly based on individual Echor03 comprised 12,561 scaffolds with a total length of 190,395,858 bp. The longest scaffold was 289,887 bp long, the contig L50 was 25,864 bp and the scaffold L50 37,262 bp. BUSCO search against the insect ortholog database identified 95.5% of single‐copy orthologs, indicating a good level of completeness.

After mapping our sample reads to the draft genome, mean read depth varied from 29 to 140 (overall mean 86 ± 29 SD) (Table S1). Of the 5,037,996 SNPs that remained in the filtered SNP dataset, 3,542,082 were shared by our ingroup samples of lice from the ringed seal subspecies and Baltic gray seal (Figure S3). The LD‐pruned SNP dataset contained 309,941 SNPs. The phylogenomic dataset consisted of 1170 50‐kb genomic windows and had a total alignment length of 57,345,636 positions, thus representing about 30% of the estimated genome size. The concatenated alignment of seven mitochondrial coding genes was 6600 bp long.

Due to the theoretical possibility of louse transfer among seal skins in tanneries or mislabelling of specimens, we determined the species identity of the host of each sequenced louse by assembling opportunistic sequencing reads that mapped to seal mitochondrial sequences (evidently originating from seal blood in the louse digestive tract). These partial assembled sequences confirmed that each louse specimen originated from the correct seal species (results not shown).

### 3.2 Host‐associated genetic differentiation and genetic diversity

PCA analysis based on the LD‐pruned SNP dataset revealed that lice from different host (sub)species cluster in different groups (Figure 2f). The first two principal components, which together explained 42.9% of the total genetic variation, mainly separated lice from Saimaa ringed seal and lice from Baltic gray seal from the less differentiated lice sampled from Arctic, Baltic and Ladoga ringed seals.

The number of polymorphic sites was 1,638,129 for lice from Arctic ringed seal, 1,508,043 for lice from Baltic ringed seal, 1,312,543 for lice from Ladoga ringed seal, 674,445 for lice from Baltic gray seal and 219,222 for lice from Saimaa ringed seal (Figure S4a). As expected, downsampling the Baltic, Ladoga and Saimaa population samples to three individuals per population led to slightly lower estimates (on average 1,210,393; 1,171,360; and 198,965 polymorphic sites, respectively; Figure S4a). The level of individual heterozygosity followed a similar pattern and was highest in lice from Arctic ringed seal (0.275–0.280 when including only unlinked variant sites and 0.0069–0.0070 when including also invariant and linked sites) and lowest in lice from Saimaa ringed seal (0.023–0.035 and 0.0006–0.0009 respectively) (Figure S4b,c). A total of 21% of the polymorphic sites were private to lice from Arctic ringed seals, 10% each to lice from Baltic ringed seals and Baltic gray seals, 8% to lice from Ladoga ringed seals, and only 1% to lice from Saimaa ringed seals (Figure S3a). Of the polymorphic variants that were present in more than one population, the largest number, 7% of all, was shared by lice from Ladoga, Baltic and Arctic ringed seals, and another 6% were shared by all populations except that from the Saimaa ringed seal (Figure S3a). Consistent with this pattern, the most fixed genetic variants were found between lice from Baltic gray seals and the rest (399,711 SNPs) and between lice from Saimaa ringed seals and the rest (31,589) (Figure S3b).

The genetic divergence in the mitochondrial COI gene between lice within louse populations from the four different ringed seal subspecies ranged from 0.0% to 0.28% (Table S2). The COI divergence between louse populations of different ringed seal subspecies was somewhat higher, with mean divergence ranging from 0.20% to 0.94%. These low divergences contrasted to mean divergences between the population of lice on the Baltic gray seal and those on the four subspecies of ringed seals, which ranged from 5.35% to 5.61%. Furthermore, the divergence between the louse population on the Baikal seal and those on other seal species was notably even higher than this, with the mean ranging from 12.55% to 12.89%.

### 3.3 Nuclear and mitochondrial phylogenies

The partitioned IQ‐TREE analyses yielded a tree topology supported by high bootstrap values (Figure 2g). As in the PCA plot, all seal louse individuals were grouped according to host seal population, with 100% bootstrap support for the monophyly of each population. Long branches leading to lice collected from Baikal seal, Baltic gray seal, and Saimaa ringed seal were supported by most gene and site trees (gCF: 87–100, sCF: 96–100). As expected, the shorter branches grouping lice from Ladoga, Baltic and Arctic ringed seals received much lower gene and site concordance factors (gCF: 10–14, sCF: 44–57) (Figure 2g and Table S3).

While the overall backbone structure of the ML phylogeny received strong bootstrap support, the relationships among ringed seal louse populations were associated with a high level of phylogenetic discordance (Figure 2g and Figure S5). The placement of lice from Ladoga ringed seal as sister to lice from Baltic ringed seal was supported by 6.2% of gene trees and 37.8% of sites (Figure S5a and Table S3). The branch joining lice from Saimaa ringed seal as sister to the Baltic Sea + Lake Ladoga clade was supported by 8.6% of the gene trees and 40.8% of sites (Figure S5b and Table S3). However, in both cases alternative topologies were generally supported by a clearly lower proportion of both gene trees and sites (Table S3).

The phylogenetic tree based on sequences of mitochondrial coding genes (Figure S6) was for the most part strongly supported and nearly identical to the nuclear tree (Figure 2g). The main discrepancy was that, in the mitochondrial tree, lice from Baltic ringed seals formed a paraphyletic group with respect to a clade consisting of lice from Ladoga ringed seal. However, this arrangement involved a very short and weakly supported branch, essentially creating a polytomy at the base of the Baltic + Ladoga clade (Figure S6). In general, long branches that had high gCFs in the nuclear tree (e.g. the branches subtending lice from Saimaa ringed seal, Baltic gray seal and Baikal seal) also had high bootstrap support in the mitochondrial tree. Conversely, short branches with low gCFs in the nuclear tree, such as the ones subtending lice from Baltic and Arctic ringed seals, were also weakly supported in the mitochondrial tree. The exception was the placement of lice from Saimaa ringed seal as sister to the clade formed by lice from Ladoga and Baltic ringed seals, which was strongly supported in the mitochondrial tree despite low gene and site concordance in the nuclear data.

### 3.4 Hybridization

**FIGURE 3:**
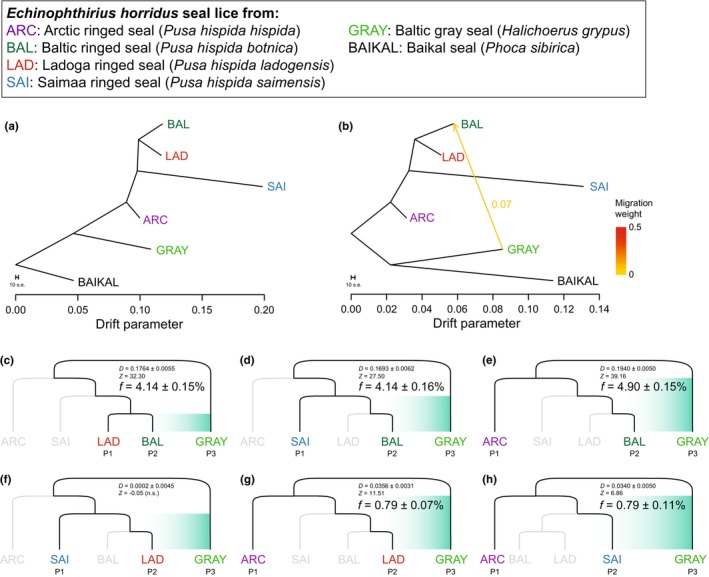
Maximum likelihood trees inferred in TreeMix based on the LD‐pruned SNP dataset while allowing (a) no migration events and (b) one migration event. Trees were rooted with lice from Baikal seal, and branch lengths reflect the amount of genetic drift. The arrow in (b) indicates the estimated direction and intensity of gene flow. For trees with more migration edges, see Figure S7. (c–h) Four‐taxon ABBA/BABA tests of introgression and estimated fractions of admixture (*f*) at different time scales: (c–e) between lice of Baltic gray and ringed seals, (f–g) between lice of Baltic gray and Ladoga ringed seals, and (h) and between lice of Baltic gray and Saimaa ringed seals. P1, P2 and P3 refer to the three populations used for the ABBA/BABA tests, values of *D* and *f* are given with their standard errors.

The TreeMix tree without migration (Figure 3a and Figure S7a) explained 99.49% of the variance in the observed covariance matrix, but positive residual covariances between lice from Baltic gray and Baltic ringed seals stood out from the rest (Figure S7b). As expected, the first migration edge was placed between these two populations, resulting in a tree that explained 99.77% of the variance in relatedness between populations (Figure 3b and Figure S7c,d), thus reaching the 99.8% threshold of explained variance suggested by the authors of TreeMix (Pickrell & Pritchard, 2012). This model inferred that 7% of the ancestry of lice from Baltic ringed seal comes from lice of Baltic gray seal (Figure 3b), while the remaining relationships among louse populations were fully consistent with the individual‐based ML tree (Figure 2g). The second and third migration edges were placed, respectively, between the ancestor of lice from Baikal seals and lice from Saimaa ringed seals, and between lice from Baltic and Arctic ringed seals (Figure S7e,g). However, fitting more than one migration edge changed the tree topology, so that lice from Ladoga ringed seal diverged before the split between lice from Baltic and Saimaa ringed seals.

We examined the rates of gene flow between lice of ringed and gray seals further using ABBA‐BABA tests (Figure 3c–h). Regardless of whether lice from Ladoga, Saimaa, or Arctic ringed seal were used as P1, the tests showed significant excess of shared alleles between lice from Baltic ringed and gray seals. For the first time period, subsequent to the divergence between lice from Baltic and Ladoga ringed seals, the estimated admixture proportion (*f*) was 4.1% (Figure 3c). The same estimate of *f* was also obtained for the longer time period subsequent to the divergence between lice from Saimaa and Baltic ringed seals (Figure 3d). When considering the period subsequent to the divergence of lice from Baltic and Arctic ringed seals, the estimated admixture proportion increased only to 4.9% (Figure 3e). In tests involving lice from Ladoga and Saimaa ringed seal as recipient populations, excess ABBA over BABA was not statistically significantly different from zero (Figure 3f), or significant but with a very low estimated admixture proportion (Figure 3g,h).

### 3.5 Demographic history

Of the six models we initially tested (Models 1–6 in Figure S2 and Table 1) in fastsimcoal2, the models assuming a common ancestor of lice from Saimaa, Ladoga, and Baltic ringed seals (Models 1–3) received more support than the model in which lice from Saimaa ringed seal diverged earlier (Model 4) or the one in which Saimaa and Ladoga lice separated from an unsampled (ghost) population (Model 5). The model with the order of population splits consistent with geological history of the lakes was the best supported, but only when gene flow between lice from Baltic gray and ringed seals was taken into account (Model 6). Of the models assuming no gene flow, the best supported model was actually Model 2, which corresponds to the second discordant topology on the ML tree (i.e. the split between lice from Saimaa and Ladoga ringed seal occurred after the split of lice from the Baltic ringed seal), and Model 1 (consistent with the ML tree and the geological history of the lakes) was the second best. Adding a single admixture event between lice from Baltic gray and ringed seals (Model 6) greatly improved the fit of Model 1, which increased further when single admixture was replaced by continuous (Model 7) or ancient (Model 8) gene flow (Figure S2 and Table 1). Of these, the model with ancient gene flow was better supported than the one with continuous gene flow, so we used Model 8 for parameter estimation.

**TABLE 1:** Results of model selection with fastsimcoal2.

Model	Number of parameters	Log‐likelihood	ΔAICa^a^	Relative likelihood^b^
Model 1	12	−426,973.34	53,802.65	~0
Model 2	12	−426,918.12	53,548.33	~0
Model 3	12	−427,035.19	54,087.45	~0
Model 4	11	−430,337.06	69,291.13	~0
Model 5	13	−428,882.81	62,598.09	~0
Model 6	14	−423,272.95	36,765.72	~0
Model 7	15	−415,796.25	2336.25	~0
Model 8	16	−415,288.51	–	–

*Note*: For a graphical representation of the models, see Figure S2.

^a^ Difference in Akaike information criterion relative to the best‐fitting model.

^b^ Relative likelihood of models based on AIC.

**FIGURE 4:**
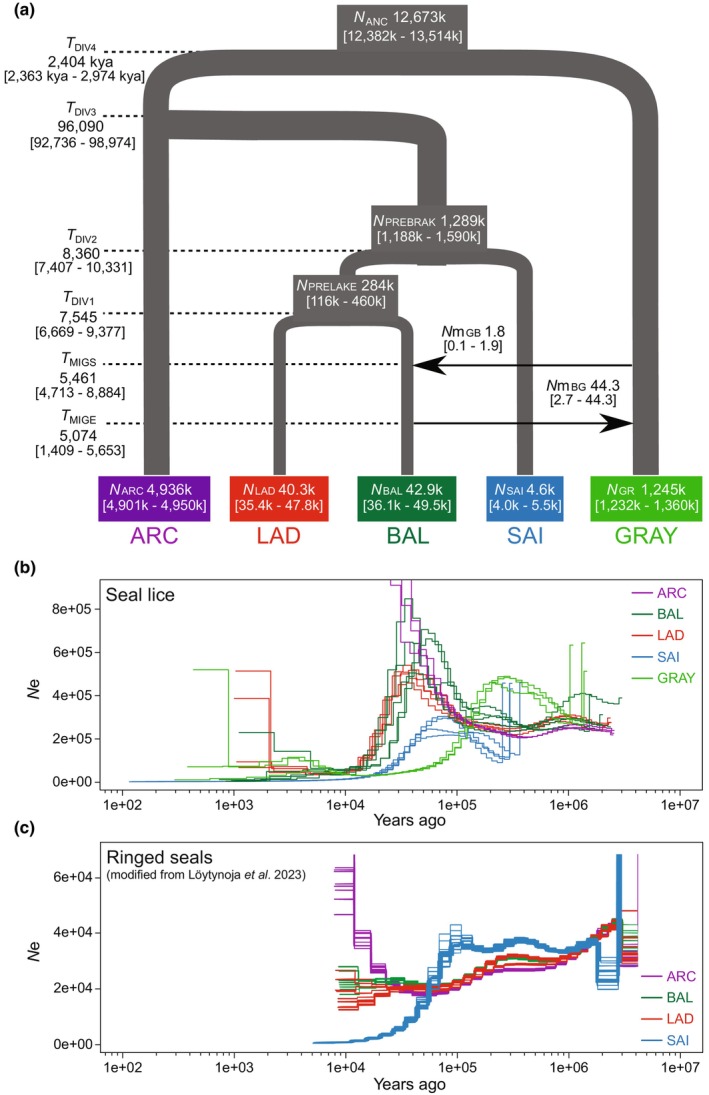
Demographic histories of seal louse populations on gray seal and four subspecies of ringed seals based on (a) the most supported model in fastsimcoal2 and (b) PSMC analyses. In (a), estimates represent divergence times (*T*, in years), effective population sizes (*N*) and numbers of migrants (*N*_m_) per generation. Numbers in brackets are 95% confidence intervals obtained through non‐parametric bootstrapping. In (b), separate lines represent individual seal lice. (c) Corresponding long‐term demographic reconstructions for the focal ringed seal subspecies estimated by Löytynoja et al. (2023) using MSMC2 analyses of seal genome resequencing data (redrawn from the original article, which was published under a CC‐BY 4.0 licence). Louse and host populations are abbreviated as in Figure 3.

According to the ML point estimates, the lineage leading to the extant louse populations of Baltic, Ladoga, and Saimaa ringed seals diverged from lice of Arctic ringed seal 192,179 generations ago, which corresponds to 96,090 [95% CI 92,736–98,974] years assuming two louse generations per year (Figure 4a). Saimaa ringed seal lice diverged from the common ancestor of lice from Baltic and Ladoga ringed seals 8360 [7407–10,331] years ago, while the latter two populations became separated 7545 [6669–9377] years ago. The simulation revealed very wide variation in estimated current effective sizes of the focal louse populations (Figure 4a). The smallest effective population size was estimated for lice of Saimaa ringed seal, at around 4600 individuals, while estimates were nearly an order of magnitude higher for lice of Baltic and Ladoga ringed seals. The estimated effective population sizes for lice of Baltic gray seals and Arctic ringed seals were substantially higher (around 1.2 and 4.9 million respectively).

Based on the estimate from the coalescent simulation, the lineages of lice infesting gray and ringed seals were separated around 2.4 million years ago (Figure 4a). Despite wide variance in estimates of the number of migrants (Figure 4a), we found evidence of asymmetrical gene flow, occurring mainly in the direction from lice of the Baltic ringed seal to those of the Baltic gray seal, starting around 5500 years ago and persisting for about 400 years.

The PSMC plots generated from individual seal louse genomes showed relatively consistent long‐term *N*_e_ trajectories comparing individuals belonging to the same population, but clear differences across populations (Figure 4b). The population‐size trajectory of lice on gray seals peaks around 200,000–300,000 years ago, but also shows a small transient increase around 3000–4000 years ago. All louse populations associated with ringed seals exhibit a concurrent increase that commences around 200,000 ago and then intensifies at about 100,000 years ago. The population peak of Saimaa ringed seal lice is lowest, and the population size then decreases earlier than in the three other populations. The *N*_e_ trajectories of lice associated with Baltic and Ladoga ringed seals undergo a rapid decline between 10,000 and 30,000 years ago. By contrast, the effective population size of lice associated with the Arctic ringed seal continues to increase and becomes inestimable towards the recent.

## 4 DISCUSSION

Genetic analyses of rapidly‐evolving host‐specific parasites can offer improved resolution for inferring population differentiation and past demographic events in their hosts (Ascunce et al., 2023; Criscione et al., 2006; Šimková et al., 2022; Whiteman et al., 2007). The impetus for the present work was to explore whether the genomes of seal‐associated lice (*Echinophthirius horridus*) could shed light on how and when ringed seals colonized the Baltic Sea basin and large postglacial lakes in its vicinity, and how the population sizes of ringed and gray seals have fluctuated through the Pleistocene. As our main analysis, we used model selection and parameter estimation in a coalescent framework, complemented by PSMC‐based inference of changes in effective population sizes over longer time periods. Combining genome‐wide data with demographic modelling represents a powerful strategy for testing alternative hypotheses about historical drivers of existing population structure and genetic diversity (Nadachowska‐Brzyska et al., 2016; Salmona et al., 2017; Zhou et al., 2014). However, despite the numerous interesting questions that can be quantitatively addressed, demographic modelling has thus far only rarely been applied to parasite species (Angst et al., 2022; Ascunce et al., 2023; Cooper et al., 2020; Hecht et al., 2018; Techer et al., 2022).

### 4.1 Population‐genomic structuring and diversity are correlated in seal lice and their hosts

Many louse taxa have become important model systems for research on specialization, speciation and coevolution, because lice tend to have narrow host ranges (Johnson et al., 2011; Kim, 2006), frequently exhibit genetic differentiation across host taxa (Demastes et al., 2012; Johnson et al., 2021; Sweet et al., 2018), and sometimes even show partial phylogenetic congruence with their hosts (Hughes et al., 2007; Johnson et al., 2022). In the case of *E. horridus*, we found distinct differentiation in nuclear (Figure 2f,g) as well as mitochondrial (Figure S6) genomes among specimens collected from different seal species and populations (for taxonomic implications, see Text S1). The overall pattern of genome‐wide variation within and among our focal northern European louse populations (Figure 2f) is strikingly similar to that found in their seal hosts (Löytynoja et al., 2023; see also Nyman et al., 2014; Peart et al., 2020). Among lice as well as ringed seals, the population from Lake Saimaa is the most differentiated from the others (Figure 2f), having the highest proportion of fixed genetic differences (Figure S3b) and showing remarkably reduced genetic diversity (Figure S4).

Interestingly, however, the general gradient of genetic diversity across populations appears to be more pronounced in the lice than in their hosts. Studies based on various nuclear (Martinez‐Bakker et al., 2013; Nyman et al., 2014; Palo et al., 2001; Peart et al., 2020; Stoffel et al., 2018) and mitochondrial (Heino et al., 2023; Palo, 2003; Valtonen et al., 2012; see also Martinez‐Bakker et al., 2013) markers in the seals have consistently indicated that genetic diversity is nearly equal in Arctic and Baltic ringed seals, and that the Ladoga ringed seal is only slightly less variable than the latter. Recently, Löytynoja et al. (2023) somewhat surprisingly reported lower genome‐wide heterozygosity and nucleotide diversity in Arctic ringed seals than in the Baltic and Ladoga subspecies, but they also cautioned that the result may reflect lower mean sequencing coverage in samples from the Arctic population (see also Kardos & Waples, 2024; Rosing‐Asvid et al., 2023). In our louse data, both the number of polymorphic sites and individual‐level heterozygosity were higher in the population associated with the Arctic ringed seal than in those on the Baltic and landlocked subspecies (Figure S4). In the case of lice on Baltic gray seal, the estimates of genetic diversity appear low in relation to the current host population size of around 55,000 individuals (Carroll et al., 2024). On the other hand, this may be the case also for the gray seals themselves (Peart et al., 2020; Yakupova et al., 2023), suggesting that the genetic uniformity of gray seals and their lice is connected to demographic events at deeper timescales in their history (see below).

### 4.2 Louse demography reflects postglacial changes in northern European geology and seal host populations

The northward retreat of the Scandinavian Ice Sheet commenced about 17,000 years ago (Tylmann & Uścinowicz, 2022; Ukkonen, 2002). This led to the formation of the current Baltic Sea through a succession of alternating freshwater and marine stages reflecting the net effect of global sea‐level rise caused by the disappearance of continental ice sheets and isostatic bedrock rebound in previously glaciated regions (Figure 2a–e). The rising bedrock also created large lakes that harbour typical marine species as glacial relicts to this day, including crustaceans (Särkkä et al., 1990), fish (Kontula & Väinölä, 2003), and – in the case of lakes Saimaa and Ladoga – even seals (Ukkonen et al., 2014). Although the geological history of the Baltic Sea and large postglacial lakes as such is well known, pinpointing how and when lakes Saimaa and Ladoga were colonized by ringed seals has proven complicated (Heino et al., 2023; Löytynoja et al., 2022; Nyman et al., 2014; Palo, 2003). The problems apparently arise from the occurrence of multiple sequential population splits within in a short time frame in relation to the genetic variability and generation time of ringed seals.

In contrast to the most recent results concerning ringed seals by Löytynoja et al. (2022, 2023), our analyses based on multiple different datasets and statistical approaches consistently supported the traditional hypothesis (Davies, 1958; Ukkonen, 2002) that the Baltic and landlocked populations are monophyletic (Figures 2g, 3a,b, 4a, Figures S6 and S7), and that the two postglacial lakes were colonized in the order in which they were formed. As in ringed seals (Löytynoja et al., 2022), the rapid succession of population divergences is seen as low gene and site concordance factors even for branches that receive strong bootstrap support on the phylogenetic trees (Figure 2g, Figure S5, Table S3). The improved resolution of the louse phylogeny in relation to the phylogenetic tree of their seal hosts is most likely explained by the shorter generation time and higher mutation rate of lice (Johnson et al., 2014), which has provided more opportunity for variants to arise and go to fixation by drift between population splits.

Furthermore, our best‐fitting fastsimcoal2 reconstruction of the demographic history of louse populations from Saimaa and Ladoga ringed seals (Figure 4a) is consistent with the presumed timeline of lake formation (Figure 2a–e). The oldest known ringed seal fossils from the current Baltic Sea coast are dated to around 10,400–10,200 years ago (Sommer & Benecke, 2003; Ukkonen et al., 2014). Our estimated origination time of lice of Saimaa ringed seals (Figure 4a) closely matches the separation of the Lake Saimaa complex from the Baltic basin approximately 9500–8000 years ago (Schmölcke, 2008; Ukkonen, 2002). Based on our analyses, lice associated with Ladoga ringed seals diverged from the Baltic source population roughly a thousand years later. While the main basin of Lake Ladoga was initially separated at about the same time as Lake Saimaa, geological evidence points towards the existence of a broad connection with the Baltic Sea until about 4000 years ago (Saarnisto, 2011; Ukkonen, 2002). This so‐called Heinjoki paleostrait has been put forth as a potential explanation for the weak genetic differentiation between Ladoga and Baltic ringed seals (Nyman et al., 2014), but our louse results suggest that movement of seals to and from Lake Ladoga was restricted already well before the estimated closing of the paleostrait.

The scaling of these estimates in absolute time is naturally influenced by the mutation rate (Martin & Höhna, 2018; Salmona et al., 2017), for which direct estimates from seal lice are not available. Like many corresponding studies on non‐model insects (Walton et al., 2021; Wang et al., 2024; Zhang et al., 2021), we therefore resorted to using an estimate from *Drosophila* (Keightley et al., 2009) in our demographic model. However, available evidence points to relatively similar rates of mutation across insect taxa (Liu et al., 2017). More importantly, the ages of the two youngest splits in our best‐fitting model (Figure 4a) are compatible with constraints imposed by geology: due to a slow postglacial change in the tilt of the rising bedrock, the outlet of Lake Saimaa gradually shifted from the northwest towards the south and finally abruptly towards the southeast c. 6000 years ago (Saarnisto, 2011). The new outlet (Vuoksi River) still has a series of steep rapids that would have prevented seal colonization after this hydrological shift. Furthermore, it is highly unlikely that the divergence between Baltic and Ladoga ringed seals would have occurred before the formation of Lake Ladoga. The fact that our time estimates fall within the time window allowed by these geological constraints points to the conclusion that the mutation rate and generation time applied in our models are approximately correct.

Reflecting the aforementioned levels of genetic diversity, the estimated effective population sizes of the focal seal louse lineages differ by three orders of magnitude (Figure 4a). Our estimates are correlated with – but in most cases substantially higher than – census population sizes (Figure 1c) as well as *N*_e_ estimates published for their seal hosts (Nyman et al., 2014; Palo, 2003; Stoffel et al., 2018). That louse *N*_e_ would exceed host *N*_e_ is as such not surprising, because each seal individual can harbour many louse individuals (Herzog, Wohlsein, et al., 2024). Even when considering this, particularly the *N*_e_ of 4600 estimated for lice from Lake Saimaa, the *N*_e_ of over 40,000 for lice from Lake Ladoga and *N*_e_ of over one million for lice of Baltic gray seals appear excessive, because microsatellite‐based estimates of current effective population sizes of their hosts range from a few tens in the severely bottlenecked Saimaa ringed seal to a few hundred in the Ladoga ringed seal and somewhere between ten and twenty thousand in the Baltic gray seal (Klimova et al., 2014; Stoffel et al., 2018; Valtonen et al., 2014). However, it is known that effective population size may be inflated by population structure (Charlesworth, 2009), which is likely to occur in lice as a result of their existence as transient, partially isolated infrapopulations on individual hosts (Virrueta Herrera et al., 2022). For lice of Baltic gray seals, the relatively high *N*_e_ estimate may also reflect past introgression from the louse population of Baltic ringed seals, which was detected by several different statistical approaches (Figures 3b–e and 4a). While the inferred direction of gene flow differs between our TreeMix (Figure 3b) and fastsimcoal2 (Figure 4a) results, several points suggest that the latter are more likely to be correct. Incorrectly oriented migration edges was one of the main errors found in the simulation‐based TreeMix tests of Pickrell and Pritchard (2012), and the TreeMix algorithm models admixture between populations as occurring at a single time point. In our fastsimcoal2 simulations, such a scenario was found to be less likely than models with continuous or ancient gene flow, and estimates of the latter indicated asymmetric gene flow from lice of Baltic ringed seals to those of gray seals.

It should also be noted that published *N*_e_ estimates for the seal hosts vary greatly depending on the marker and method used and the time scale considered. In the case of lice of Saimaa and Baltic ringed seals, our estimates are more reconcilable with long‐term seal *N*_e_ estimates based on SNPs (Peart et al., 2020) and microsatellites and/or mtDNA control‐region sequences (Nyman et al., 2014; Palo et al., 2001; Valtonen et al., 2012). The explanation for the apparently high *N*_e_/*N*_c_ ratios in these seal louse populations is therefore likely to be the same as the one proposed by Peart et al. (2020) for their hosts, that is, retention of residual variation from much larger ancestral populations. However, we note that the *N*_e_ estimate of Peart et al. (2020) for the Saimaa ringed seal was only circa 40% lower than the (near‐equal) estimates for Baltic and Arctic ringed seals, meaning that our louse‐based estimates are more in line with the presumed postglacial relative census sizes of these subspecies.

### 4.3 Long‐term demographic trajectories of seal lice show imprints of Pleistocene and Holocene climatic fluctuations

Our PSMC analyses based on genome sequences of individual seal lice revealed clear differences in long‐term population‐size trajectories among louse populations associated with different seal (sub)species (Figure 4b) as well as intriguing correspondence to prior estimates from their hosts (Löytynoja et al., 2023; Yakupova et al., 2023). Many authors have noted that PSMC plots should be interpreted with caution because the scaling of both effective population size and time depends on knowledge on generation times and mutation rates (Pujolar et al., 2017; Yakupova et al., 2023). These complications are naturally compounded in comparative analyses of parasites and hosts, because the scaling parameters need to be correct on both trophic levels. Published reconstructions of demographic trajectories in seals have applied species‐specific information on generation times, but have had to rely on mutation rates estimated for polar bear (Löytynoja et al., 2023) or other marine and terrestrial mammals (Yakupova et al., 2023) for scaling. Furthermore, the fact that PSMC reconstructs *N*_e_ trajectories based on the rate of coalescence at different time intervals in the past means that estimates can be distorted by admixture, inbreeding and population substructure (Mather et al., 2020; Mazet et al., 2016; Morin et al., 2018; Teixeira et al., 2021). While our fastsimcoal2 results suggest that the mutation rate and generation time applied in our model are approximately correct (see above), it is unclear whether and how the transient yet regular infrapopulation structuring of lice (Virrueta Herrera et al., 2022) would affect estimates of population size stretching over many millennia. However, we note that lice generally present a comparative advantage because the potentially confounding parameters are expected to be relatively similar across populations.

Thus, even if considered only qualitatively, the seal louse PSMC trajectories show remarkable similarity between the lice within an individual population as compared to between populations, and suggest that long‐term variation in seal louse (and seal host) population sizes has been driven by climatic fluctuations through the late Pleistocene and the Holocene. Combined PSMC analyses of gray and ringed seals have thus far not been published. However, our finding of an earlier past population peak in lice associated with gray seals appears expected considering the differing niches and climatic preferences of gray and ringed seals, and resembles the pattern in the gray seals themselves (within the limits of uncertainty noted by Yakupova et al. (2023)). The main population peak in lice on gray seals was likely driven by the warmer climate of the Eemian interglacial between 130 and 100 kya (Stein et al., 2017) and possibly preceding middle Pleistocene interglacials (Hughes et al., 2020), which would have made a large part of the Arctic Ocean suitable for inhabitation by gray seals. As shown by the hindcast estimate of Boehme et al. (2012), when the global climate grew gradually cooler from the beginning of the last glacial period around 100 kya, the advancing North American and northern European ice sheets would have forced gray seals southward. At the same time, the freezing of the northern Atlantic Ocean would have separated the overall population into two subpopulations inhabiting the western and eastern sides of the Atlantic. More importantly, the same paleoenvironmental reconstruction showed that the lowering of global sea levels by up to 130 m during the last glacial maximum (LGM) may have reduced the extent of productive continental shelf seas (which support the highest densities of gray seals) by up to 97%. Our finding of a small recovery in gray seal louse populations during the last 5000 years is partly consistent with genetic signatures of a population expansion in the Baltic gray seal population during the late Holocene (Ahlgren et al., 2022; Fietz et al., 2016; Klimova et al., 2014). On the other hand, the timing of the peak overlaps with our estimate of the time window of substantial introgression from lice of Baltic ringed seals (Figure 4). The apparent recovery could therefore reflect hybridization with lice of Baltic ringed seals during an extended mid‐Holocene period of low gray seal abundance but high and widespread ringed seal occurrence in the Baltic fossil record (Ahlgren et al., 2022; Ukkonen et al., 2014).

More direct demographic comparisons are possible for the seal louse populations associated with ringed seals (Figure 4b,c). Notably, although louse and host trajectories show evidence for parallel changes especially in the Arctic, Baltic and Ladoga subspecies, the timing of events appears slightly shifted towards the recent in the seals. Part of the discrepancy could result from the use of the polar bear mutation rate in the ringed seal MSMC2 analyses by Löytynoja et al. (2023). Applying the lower seal‐specific mutation‐rate estimates of Peart et al. (2020) should stretch the ringed seal trajectories backward in time and thereby improve the correspondence between louse and host estimates. Given that the order and time frame of population splits in our results shows closer correspondence to geological constraints (see above), we tentatively consider our temporal scaling more reliable.

As would be expected for parasites of hosts that are dependent on ice and snow for reproduction (Lone et al., 2019), the population trajectories of lice associated with ringed seals enter a period of rapid growth at the beginning of the last glacial period around 100 kya, concurrently with the main decline in lice of gray seals (Figure 4b). However, the peak is transient in lice of Baltic, Ladoga, and Saimaa ringed seals, in which the trajectories turn into decline approximately 30 kya. While such downward turns at the height of the LGM may seem counterintuitive at first sight, concurrent population declines have recently been demonstrated for many Arctic cetaceans (Skovrind et al., 2021; Westbury et al., 2023). Here it is important to notice that ringed seal densities are highest along marginal ice zones above highly productive continental shelf areas (Lone et al., 2019). Therefore, although the ringed seal distribution most likely did not shift as far south as that of the gray seal during the LGM, ringed seals would still have been adversely affected by the loss of shallow continental shelf seas demonstrated by the paleoenvironmental reconstructions by Boehme et al. (2012), Foote et al. (2013) and Skovrind et al. (2021).

Why, then, do the long‐term population trajectories of neither Arctic ringed seals (Figure 4c) nor their lice (Figure 4b) exhibit a parallel decrease during the LGM, and instead exceed the scale before the beginning of the Holocene around 10,000 years ago? As such, an increasing trend through the Holocene would not be unexpected, because post‐Pleistocene population recoveries or expansions have been found in many Arctic marine mammals (Foote et al., 2013; Louis et al., 2020; Skovrind et al., 2021; Westbury et al., 2019). As shown by Cabrera et al. (2022), these responses were in many cases most likely driven by increased availability of important prey species in productive continental shelf seas, which were resubmerged as a result of the melting of continental ice sheets. Our analyses suggest that the populations of lice of Baltic and landlocked ringed seals could not respond as strongly to the increasingly mild climate, because by the early Holocene their hosts were already confined to their respective water bodies.

However, the population increases in both Arctic ringed seals and their lice appear too strong and too early to be explained by post‐Pleistocene climatic amelioration alone. Instead, we propose that broader consideration of Pleistocene climatic patterns and geology in the Arctic may provide clues. Of importance here is that our fastsimcoal2 simulations indicate that the ancestor of the lice from Baltic, Ladoga and Saimaa ringed seals was separated from the ancestor of lice of Arctic ringed seals nearly 100 kya (Figure 4a). The emergence of this structuring, with a period of diminishing gene flow, could in fact partly explain the apparent parallel rise in the effective sizes of all louse populations associated with ringed seals (cf. Cahill et al., 2016; Morin et al., 2018). While our 96‐ky age estimate for the split conflicts with the ‘standard model’ of post‐Pleistocene separation Baltic and Arctic ringed seals (Nyman et al., 2014; Palo, 2003; Schmölcke, 2008; Ukkonen, 2002), it is consistent with the fact that the Baltic ringed seal is genetically nearly as distant from the Arctic ringed seal as is the recently found Kangia ringed seal population, for which Rosing‐Asvid et al. (2023) estimated an age of over 200,000 years (see their Figure 3b). Therefore, the separation of the Arctic lineage and the common ancestor of Baltic, Ladoga and Saimaa ringed seals could have commenced already at the onset of the last (Weichselian) glaciation, which led to the formation of continental ice sheets in North America, Greenland, Iceland, Western Europe and Western Siberia (Hughes et al., 2013), and year‐round covering of a large part of the Arctic Ocean by up to 1‐km thick ice shelves (Jakobsson et al., 2014). In North America, Pleistocene ringed seal fossils are found thousands of kilometres south of their current range along the Pacific (Harington, 2008) and Atlantic coasts (Feranec et al., 2014). On the eastern side of the Atlantic, the main distribution of ringed seals is likely to have been to the west and south of the British Isles, along coastlines that are now below sea level. Fossils from these regions are therefore unavailable, but the occurrence of ringed seals outside their current range is confirmed by fossil remains from the Danish Straits dated to approximately 45,000 years ago (Ukkonen et al., 2014). Throughout most of the last glacial period, the separation of ringed seals of the Atlantic and Pacific Oceans would have been completed by the Bering land bridge between eastern Siberia and Alaska (Jakobsson et al., 2014; Praetorius et al., 2023).

Based on the above, we suggest that the PSMC population trajectory of lice of Arctic ringed seals as well as their hosts reflects postglacial fusion of populations that had been isolated after the end of the Eemian interglacial – a possibility initially proposed by Davies (1958) on the basis of distributions of morphologically defined ringed seal subspecies across the Holarctic. The Baltic Sea basin became colonized by eastern Atlantic ringed seals while they followed the northward retreat of the Scandinavian Ice Sheet at the end of the Pleistocene. The continued warming of the climate then separated the ancestral population in the Baltic Sea from the one inhabiting the North Sea coast of Norway. When the limit of wintertime sea ice continued to move northward, the population on the North Sea coast merged with the likewise northward‐moving western Atlantic population and, eventually, with seals arriving in the Arctic from the Pacific Ocean coastline. Therefore, the population explosion seen in the PSMC trajectories of Arctic seal louse and ringed seal populations would reflect a combination of (i) a real increase in the number of individuals in more benign environmental conditions and increased extent of continental shelf seas, and (ii) admixture of lineages that had been separated through most of the last glacial period. Particularly the demographic trajectory of Arctic ringed seal lice shows resemblance to artefactual population explosions found in simulation studies creating artificial ‘pseudohybrid’ genomes using data from distantly related populations (Cahill et al., 2016; Carroll et al., 2021; Morin et al., 2018). While such a scenario may seem speculative, similar Holocene fusions of Atlantic and Pacific stocks within the Arctic Ocean have also been suggested for bowhead whales (Foote et al., 2013) and belugas (Skovrind et al., 2021). Fusion of Pacific and Atlantic ringed seals seems inevitable considering that the species is highly mobile and currently forms a near‐panmictic population throughout its Arctic range (Lang et al., 2021; Martinez‐Bakker et al., 2013; Rosing‐Asvid et al., 2023).

## 5 CONCLUSIONS

Advanced sequencing technologies, computing power and statistical methods now offer unprecedented opportunities for using genomes of rapidly evolving specialist parasites as proxy markers for understanding the ecology and evolution of their hosts (Nieberding & Olivieri, 2007; Thorn et al., 2023) and as a general tool of inference in conservation biology (Gagne et al., 2022; Gupta et al., 2020; Whiteman & Parker, 2005). The breadth of possibilities is nicely illustrated by our study and those of Leonardi et al. (2019) and Virrueta Herrera et al. (2022), which in combination demonstrate how genomic analyses of seal lice can be applied to tackle research questions spanning widely different spatial, temporal and taxonomic scales.

When it comes to population‐specific genetic diversities, among‐population similarities and demographic histories, *Echinophthirius horridus* seal lice exhibit patterns that are in some cases strikingly similar to prior findings from their hosts. At the same time, seal louse genomes offer improved resolution with respect to the timing and sequence of divergence events. Notably, our results support the traditional hypothesis of stepwise colonization of the Baltic Sea and postglacial lakes during and after the disappearance of the Scandinavian Ice Sheet (Davies, 1958; Ukkonen, 2002). Over longer time scales, demographic patterns in seal lice show connections to the climatic history of the Pleistocene as well as intriguing parallels with population fluctuations, divergences, and fusions that have been suggested for Arctic cetaceans. Many questions remain, however, and follow‐up studies should aim at broader and denser sampling of populations, and should especially strive towards integrated analyses of genomic data from seals, seal lice and other seal‐associated parasites. Clearly, the full potential of parasite genomics in basic and applied biological research is yet to be realized.

## AUTHOR CONTRIBUTIONS

T.N., L.S., K.P.J., M.K. and E.Y. conceived the study. E.Y., M.K., T.N., E.A., V.A., O.R. and A.R.‐A. obtained samples. L.S., K.P.J. and S.V.H. collected the data. L.S. analysed the data. T.N., E.Y., M.K. and L.S. obtained financial support for the project. L.S. and T.N. wrote the manuscript, and all authors contributed to editing the manuscript.

## CONFLICT OF INTEREST STATEMENT

The authors declare no conflict of interest.

## OPEN RESEARCH BADGES

This article has earned an Open Data badge for making publicly available the digitally‐shareable data necessary to reproduce the reported results. The data is available at https://doi.org/10.5281/zenodo.11031997.

## BENEFIT‐SHARING STATEMENT

Benefits from this research accrue from presenting information on the biology of many endangered host–parasite systems and the sharing of our data and results on public databases as described above. This study complies with laws governing handling of endangered animals (see Section 2).

## Supporting information


Appendix S1.

## Data Availability

Raw sequence reads are deposited in the NCBI Sequence Read Archive (SRA) under BioProject accession numbers PRJNA490903, PRJNA490904, PRJNA490905, PRJNA490906, PRJNA490908, and PRJNA1103082. SNP files and other processed input files as well as scripts for all analyses are available on Zenodo (https://doi.org/10.5281/zenodo.11031997).
