# Supplementary material for: Population genomics of seal lice provides insights into the postglacial history of northern European seals
Source: Mol Ecol. 2024 Sep 9;33(20):e17523. doi: 10.1111/mec.17523 (PMC13084992; doi:10.1111/mec.17523)
Supplement: Supplementary file 1 — Appendix S1. [file MEC-33-e17523-s001.pdf]

MOLECULAR ECOLOGY

Supporting information for:

Population genomics of seal lice provides insights into the postglacial history of northern European seals

Ludmila Sromek, Kevin P. Johnson, Mervi Kunnasranta, Eeva Ylinen, Stephany Virrueta Herrera, Elena Andrievskaya, Vyacheslav Alexeev, Olga Rusinek, Aqqalu Rosing-Asvid, and Tommi Nyman

Text S1. Notes on the taxonomy of *Echinophthirius horridus*.

Figure S1. Workflow used during the production of the different nuclear population-genomic and phylogenomic datasets used in the analyses.

Figure S2. Schematic illustrations of the eight hypothetical demographic models tested in fastsimcoal2. Details of each model (template and parameter estimation files with all defined parameters and their search ranges) are available in the file package deposited on Zenodo (see Data accessibility statement). Populations are abbreviated as in Figure 3.

Figure S3. Venn diagrams depicting SNP sharing among the five focal seal louse populations. (a) Sharing of 3,108,959 polymorphic sites, (b) sharing of 433,123 fixed sites. Populations are abbreviated as in Figure 3.

Figure S4. Genetic diversity in lice collected from the five focal northern European seal populations. (a) Number of polymorphic sites in each population estimated from the filtered SNP dataset. The black portions of the bars of the Baltic, Ladoga, and Saimaa populations show mean estimates based on all possible subsamples of three individuals. (b) distributions of individual heterozygosity estimated from the LD-pruned SNP dataset. (c) distributions of individual heterozygosity estimated from the intermediate dataset of filtered genotypes containing both variant and invariant sites. Populations are abbreviated as in Figure 3.

Figure S5. Proportions of concordant and discordant gene trees for different alternative hypotheses concerning phylogenetic relationships among seal lice from the focal northern European ringed seal subspecies.

Figure S6. ML phylogenetic tree for 22 seal louse individuals calculated based on sequences of seven mitochondrial protein-coding genes. Numbers above branches are ultrafast bootstrap proportions (only values >95% shown). The tree was rooted based on the results of the nuclear phylogenomic analyses (Figure 2g).

Figure S7. Estimates of phylogenetic relationships and gene flow among lice from six northern European seal (sub)species based on TreeMix analyses of the LD-pruned SNP dataset. (a, c, e, g) Phylogeny estimates when allowing an increasing number of migration edges, (b, d, f, h) among-population residual covariance matrix for each analysis. In (a, c, e, g), branch lengths represent the amount of genetic drift, while arrows indicate the estimated direction of gene flow and are colored according to the relative migration weights shown next to the arrows. Populations are abbreviated as in Figure 3.

Table S1. Collection data and sequencing statistics for the 22 *Echinophthirius horridus* specimens analyzed in this study.

Table S2. Between- and within-group uncorrected percent COI sequence divergence among lice sampled from different seal hosts. Numbers represent means across all pairwise comparisons of sequences, with minimum and maximum values given in parentheses.

Table S3. Concordance factor statistics for each branch of the nuclear phylogenomic tree in Figure 2g.

Text S1

Notes on the taxonomy of *Echinophthirius horridus*

Sucking lice in the family Echinophthiriidae apparently followed their hosts through the initial terrestrial–aquatic transition 23–30 million years ago (Berta et al., 2018), and currently exclusively parasitize pinnipeds and river otters (Leidenberger et al., 2007; Leonardi et al., 2021). The family consists of five genera and 13 species, most of which are presumed to occur on single pinniped species (Leidenberger et al., 2007; Leonardi et al., 2019). Leonardi and Palma (2013) listed two exceptions to the general pattern: *Antarctophthirus microchir*, which had been listed from six species of sea lions (Otariidae), and *Echinophthirius horridus*, which occurs on all northern true seals (*i.e.*, species within *Pusa*, *Phoca*, *Halichoerus*, *Erignathus*, *Cystophora*, and *Pagophilus*). However, Leonardi et al. (2019) found very deep genetic differentiation across *A. microchir* specimens collected from South American and Australian sea lions and, therefore, postulated that *A. microchir* is in fact likely to be a complex of morphologically indistinguishable, or difficult to distinguish, species.

Based on the distinct differentiation present in nuclear (Figure 2f,g) as well as mitochondrial (Figure S6) genomes among some of our focal populations, *E. horridus* is likewise likely to comprise a species complex rather than a single generalist lineage. Studies applying the standard "barcode" region of the mitochondrial COI gene in animals have frequently assumed circa 2% sequence divergence as the "barcode gap" distinguishing intra- from interspecific variation (Hebert et al., 2003; Zhang and Bu, 2022; Cheng et al., 2023). However, lice have an elevated mitochondrial substitution rate compared to other insects (Johnson et al., 2003), and recent phylogenomic studies on species-rich bird lice have instead proposed 5% uncorrected sequence divergence in full-length COI sequences as the limit for defining species (Johnson et al., 2021; Doña and Johnson, 2023). Regardless of which one of these thresholds is used, our

results indicate that lice from the four focal ringed seal subspecies (with mean interpopulation divergences between 0.2 and 0.9% in our COI sequence data) are conspecific, while those found on Baltic gray seal and Baikal seal (with over 5.2 and 12.3% divergences from lice on other seal species, respectively) likely constitute two distinct species (Table S2). Further studies on the status of these lineages are clearly warranted.

Most of the genome-wide variation within and among our focal seal louse populations (Figure 2f) is evidently explained by dispersal limitation in their hosts. Saimaa and Ladoga ringed seals are confined to their respective lakes, while the Baltic ringed seal population is separated from the southern distribution limit of the Arctic ringed seal by over two thousand kilometers of seas with ice-free winters preventing the construction of subnivean breeding lairs (Figure 1c) (Schmölcke, 2008; Kovacs et al., 2011). While the lineages of lice on gray and ringed seals most likely were separated over two million years ago (Figure 4a), several different statistical approaches revealed a low level of recent gene flow between them within the Baltic Sea (Figure 3). Hence, fusion of the louse populations of the broadly sympatric Baltic ringed and gray seals seems to be prevented by differences in the seals' habitat preferences. The most opportunities for lice to colonize new hosts occur during the breeding and molting seasons of seals, when they spend much time hauled out on ice or land (Leonardi et al., 2021). During the winter and spring, gray seals are largely absent from the coastal areas with land-fast ice (Oksanen et al., 2014) that ringed seals use for hauling out and breeding (Halkka and Tolvanen, 2017), and the solitary behavior of ringed seals (Hammill, 2009) likely enhances the isolation of their louse populations during the ice-free period. It is noteworthy that these habitat and behavioral differences between Baltic ringed and gray seals are not strong enough to cause marked host-associated genetic differentiation in three species of intestinal *Corynosoma* parasites (Acanthocephala: Polymorphidae), all of which are transmitted among individual seals through a series of intermediate crustacean and fish hosts (Sromek et al., 2023). This difference in

population-genetic patterns between parasites may reflect a general tendency for parasites with complex, indirect life histories to have low host specificity (Nieberding and Olivieri, 2007; Mazé-Guilmo et al., 2016).

References

Berta, A., Churchill, M., Boessenecker, R.W., 2018. The origin and evolutionary biology of pinnipeds: seals, sea lions, and walruses. Annual Review of Earth and Planetary Sciences 46, 203–228. https://doi.org/10.1146/annurev-earth-082517-010009

Cheng, Z., Li, Q., Deng, J., Liu, Q., Huang, X., 2023. The devil is in the details: Problems in DNA barcoding practices indicated by systematic evaluation of insect barcodes. Frontiers in Ecology and Evolution 11.

Doña, J., Johnson, K.P., 2023. Host body size, not host population size, predicts genome-wide effective population size of parasites. Evolution Letters 7, 285–292. https://doi.org/10.1093/evlett/qrad026

Halkka, A., Tolvanen, P. (Eds.), 2017. The Baltic ringed seal – An Arctic seal in European waters - WWF Finland report 36. WWF Suomi, Helsinki.

Hammill, M.O., 2009. Ringed seal *Pusa hispida*, in: Perrin, W.F., Würsig, B., Thewissen, J.G.M. (Eds.), Encyclopedia of Marine Mammals (Second Edition). Academic Press, London, pp. 972–974. https://doi.org/10.1016/B978-0-12-373553-9.00221-2

Hebert, P.D.N., Ratnasingham, S., de Waard, J.R., 2003. Barcoding animal life: cytochrome *c* oxidase subunit 1 divergences among closely related species. Proceedings of the Royal Society B: Biological Sciences 270, S96–S99. https://doi.org/10.1098/rsbl.2003.0025

Johnson, K.P., Cruickshank, R.H., Adams, R.J., Smith, V.S., Page, R.D.M., Clayton, D.H., 2003. Dramatically elevated rate of mitochondrial substitution in lice (Insecta: Phthiraptera). Molecular Phylogenetics and Evolution 26, 231–242. https://doi.org/10.1016/S1055-7903(02)00342-1

Johnson, K.P., Weckstein, J.D., Virrueta Herrera, S., Doña, J., 2021. The interplay between host biogeography and phylogeny in structuring diversification of the feather louse genus *Penenirmus*. Mol Phylogenet Evol 165, 107297. https://doi.org/10.1016/j.ympev.2021.107297

Kovacs, K.M., Lydersen, C., Overland, J.E., Moore, S.E., 2011. Impacts of changing sea-ice conditions on Arctic marine mammals. Mar Biodiv 41, 181–194. https://doi.org/10.1007/s12526-010-0061-0

Leidenberger, S., Harding, K., Härkönen, T., 2007. Phocid seals, seal lice and heartworms: a terrestrial host-parasite system conveyed to the marine environment. Diseases of Aquatic Organisms 77, 235–253. https://doi.org/10.3354/dao01823

Leonardi, M.S., Crespo, J.E., Soto, F., Lazzari, C.R., 2021. How did seal lice turn into the only truly marine insects? Insects 13, 46. https://doi.org/10.3390/insects13010046

Leonardi, M.S., Palma, R.L., 2013. Review of the systematics, biology and ecology of lice from pinnipeds and river otters (Insecta: Phthiraptera: Anoplura: Echinophthiriidae). Zootaxa 3630, 445–466. https://doi.org/10.11646/zootaxa.3630.3.3

Leonardi, M.S., Virrueta Herrera, S., Sweet, A., Negrete, J., Johnson, K.P., 2019. Phylogenomic analysis of seal lice reveals codivergence with their hosts. Syst Entomol 44, 699–708. https://doi.org/10.1111/syen.12350

Mazé-Guilmo, E., Blanchet, S., McCoy, K.D., Loot, G., 2016. Host dispersal as the driver of parasite genetic structure: a paradigm lost? Ecology Letters 19, 336–347. https://doi.org/10.1111/ele.12564

Nieberding, C.M., Olivieri, I., 2007. Parasites: proxies for host genealogy and ecology? Trends in Ecology & Evolution 22, 156–165. https://doi.org/10.1016/j.tree.2006.11.012

Oksanen, S.M., Ahola, M.P., Lehtonen, E., Kunnasranta, M., 2014. Using movement data of Baltic grey seals to examine foraging-site fidelity: implications for seal-fishery conflict

mitigation. Marine Ecology Progress Series 507, 297–308. https://doi.org/10.3354/meps10846

Schmölcke, U., 2008. Holocene environmental changes and the seal (Phocidae) fauna of the Baltic Sea: coming, going and staying. Mammal Review 38, 231–246. https://doi.org/10.1111/j.1365-2907.2008.00131.x

Sromek, L., Ylinen, E., Kunnasranta, M., Maduna, S.N., Sinisalo, T., Michell, C.T., Kovacs, K.M., Lydersen, C., Ieshko, E., Andrievskaya, E., Alexeev, V., Leidenberger, S., Hagen, S.B., Nyman, T., 2023. Loss of species and genetic diversity during colonization: Insights from acanthocephalan parasites in northern European seals. Ecology and Evolution 13, e10608. https://doi.org/10.1002/ece3.10608

Zhang, H., Bu, W., 2022. Exploring large-scale patterns of genetic variation in the COI gene among Insecta: Implications for DNA barcoding and threshold-based species delimitation studies. Insects 13, 425. https://doi.org/10.3390/insects13050425

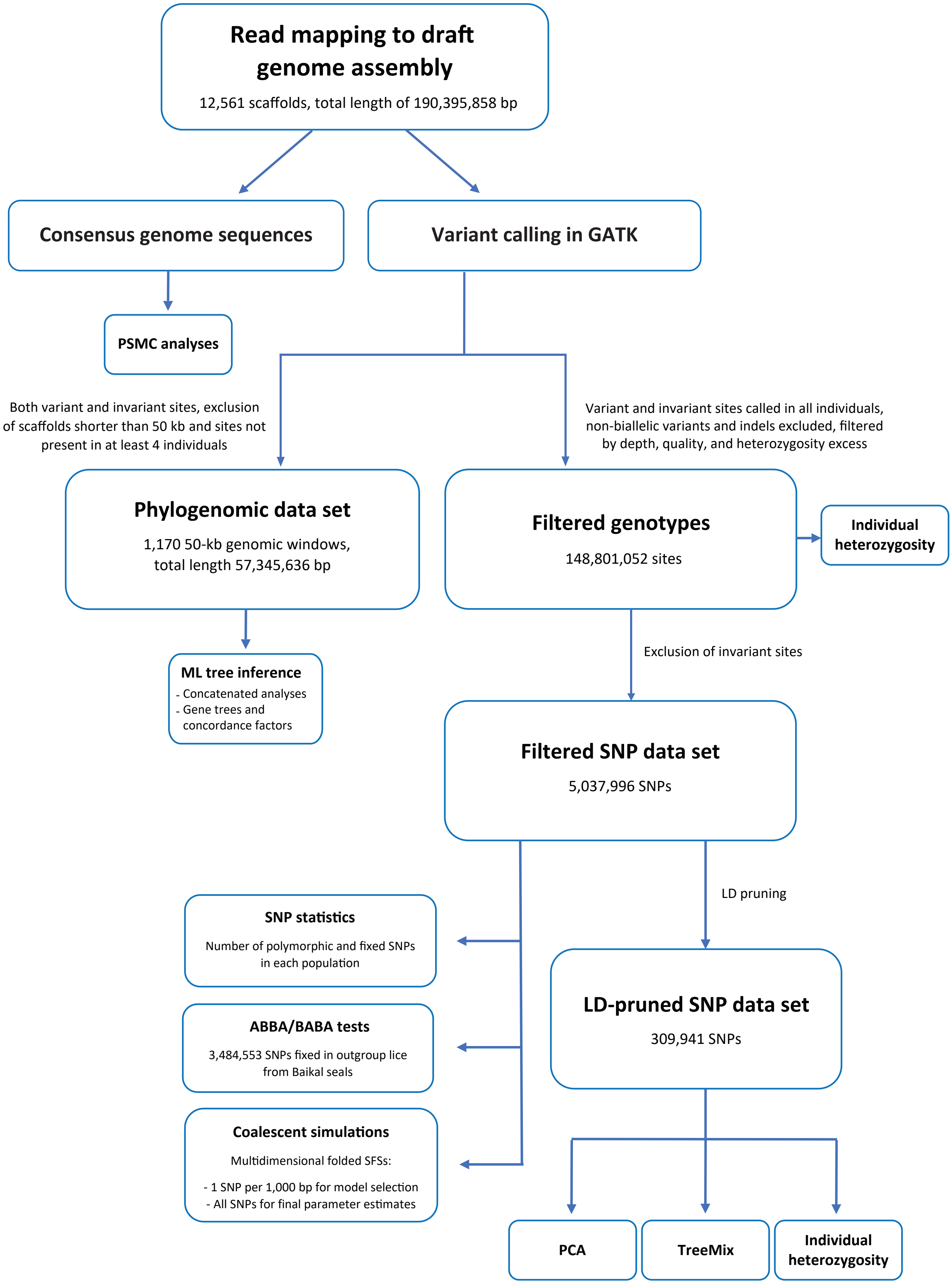

Read mapping to draft genome assembly
12,561 scaffolds, total length of 190,395,858 bp
Consensus genome sequences
Variant calling in GATK
PSMC analyses
Both variant and invariant sites, exclusion of scaffolds shorter than 50 kb and sites not present in at least 4 individuals
Variant and invariant sites called in all individuals, non-biallelic variants and indels excluded, filtered by depth, quality, and heterozygosity excess
Phylogenomic data set
1,170 50-kb genomic windows, total length 57,345,636 bp
Filtered genotypes
148,801,052 sites
Individual heterozygosity
ML tree inference
- Concatenated analyses
- Gene trees and concordance factors
Exclusion of invariant sites
Filtered SNP data set
5,037,996 SNPs
LD pruning
SNP statistics
Number of polymorphic and fixed SNPs in each population
ABBA/BABA tests
3,484,553 SNPs fixed in outgroup lice from Baikal seals
Coalescent simulations
Multidimensional folded SFSs:
- 1 SNP per 1,000 bp for model selection
- All SNPs for final parameter estimates
LD-pruned SNP data set
309,941 SNPs
PCA
TreeMix
Individual heterozygosity

Fig. S1

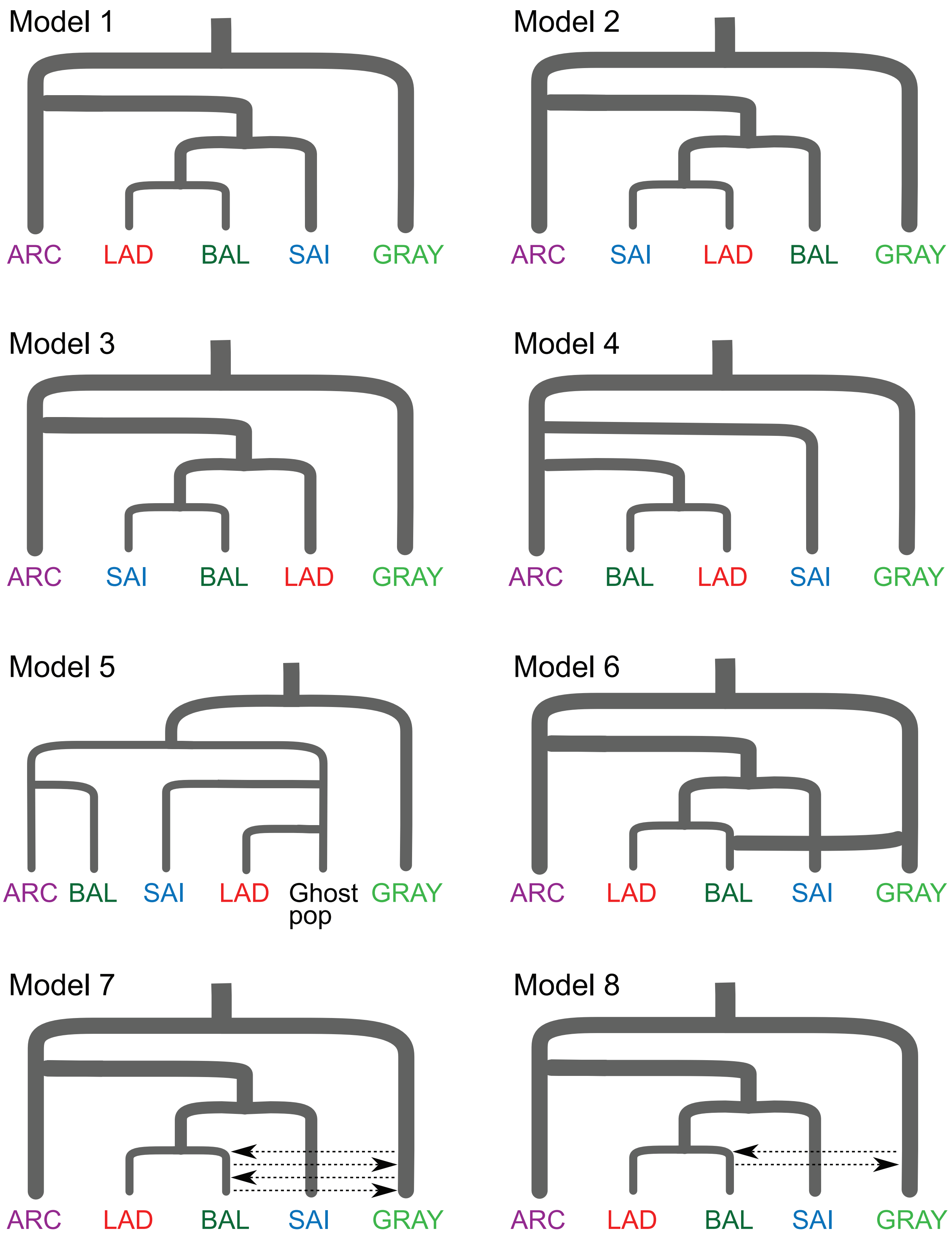
Model 1
ARC LAD BAL SAI GRAY
Model 2
ARC SAI LAD BAL GRAY
Model 3
ARC SAI BAL LAD GRAY
Model 4
ARC BAL LAD SAI GRAY
Model 5
ARC BAL SAI LAD Ghost pop GRAY
Model 6
ARC LAD BAL SAI GRAY
Model 7
ARC LAD BAL SAI GRAY
Model 8
ARC LAD BAL SAI GRAY

Fig. S2

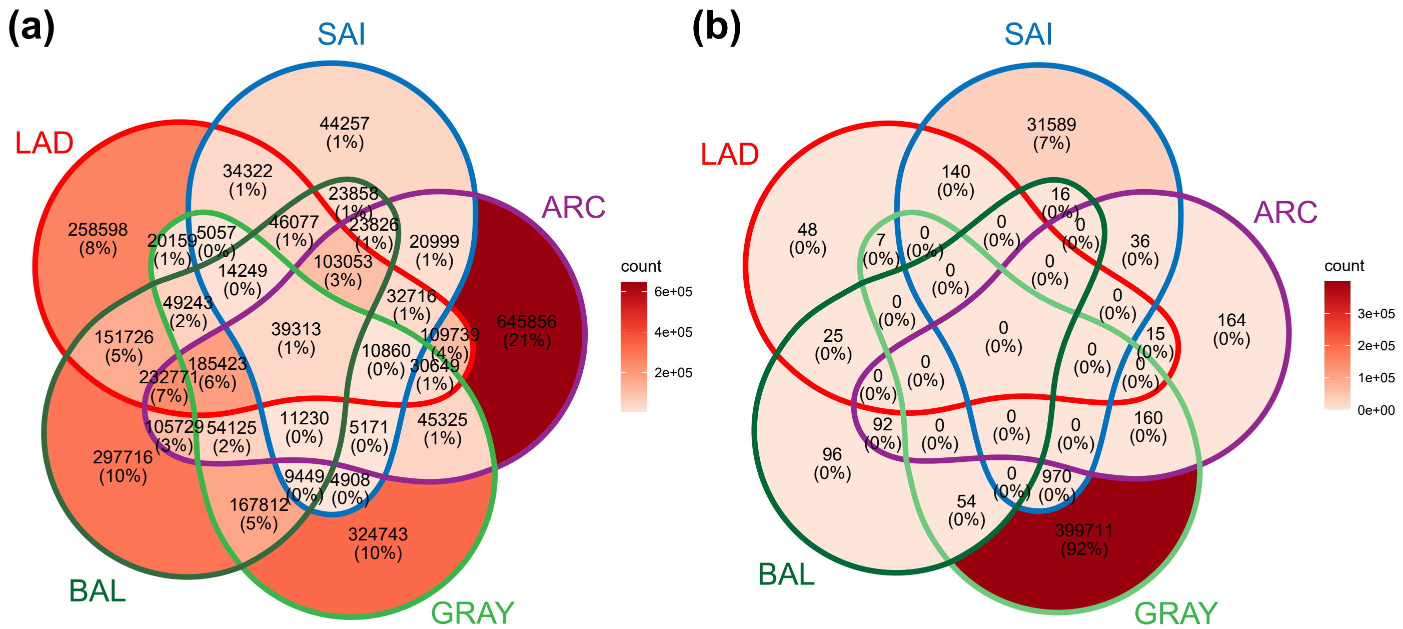
(a)
SAI
LAD
ARC
BAL
GRAY
44257
(1%)
34322
(1%)
258598
(8%)
23858
(1%)
23826
(1%)
20159
(1%)
5057
(0%)
46077
(1%)
20999
(1%)
14249
(0%)
103053
(3%)
49243
(2%)
32716
(1%)
645856
(21%)
151726
(5%)
39313
(1%)
109739
(4%)
185423
(6%)
10860
(0%)
30649
(1%)
232771
(7%)
105729
(3%)
54125
(2%)
11230
(0%)
5171
(0%)
45325
(1%)
297716
(10%)
9449
(0%)
4908
(0%)
167812
(5%)
324743
(10%)
count
6e+05
4e+05
2e+05
(b)
SAI
LAD
ARC
BAL
GRAY
31589
(7%)
140
(0%)
48
(0%)
16
(0%)
7
(0%)
0
(0%)
36
(0%)
25
(0%)
15
(0%)
164
(0%)
92
(0%)
160
(0%)
96
(0%)
54
(0%)
970
(0%)
399711
(92%)
count
3e+05
2e+05
1e+05
0e+00

Fig. S3

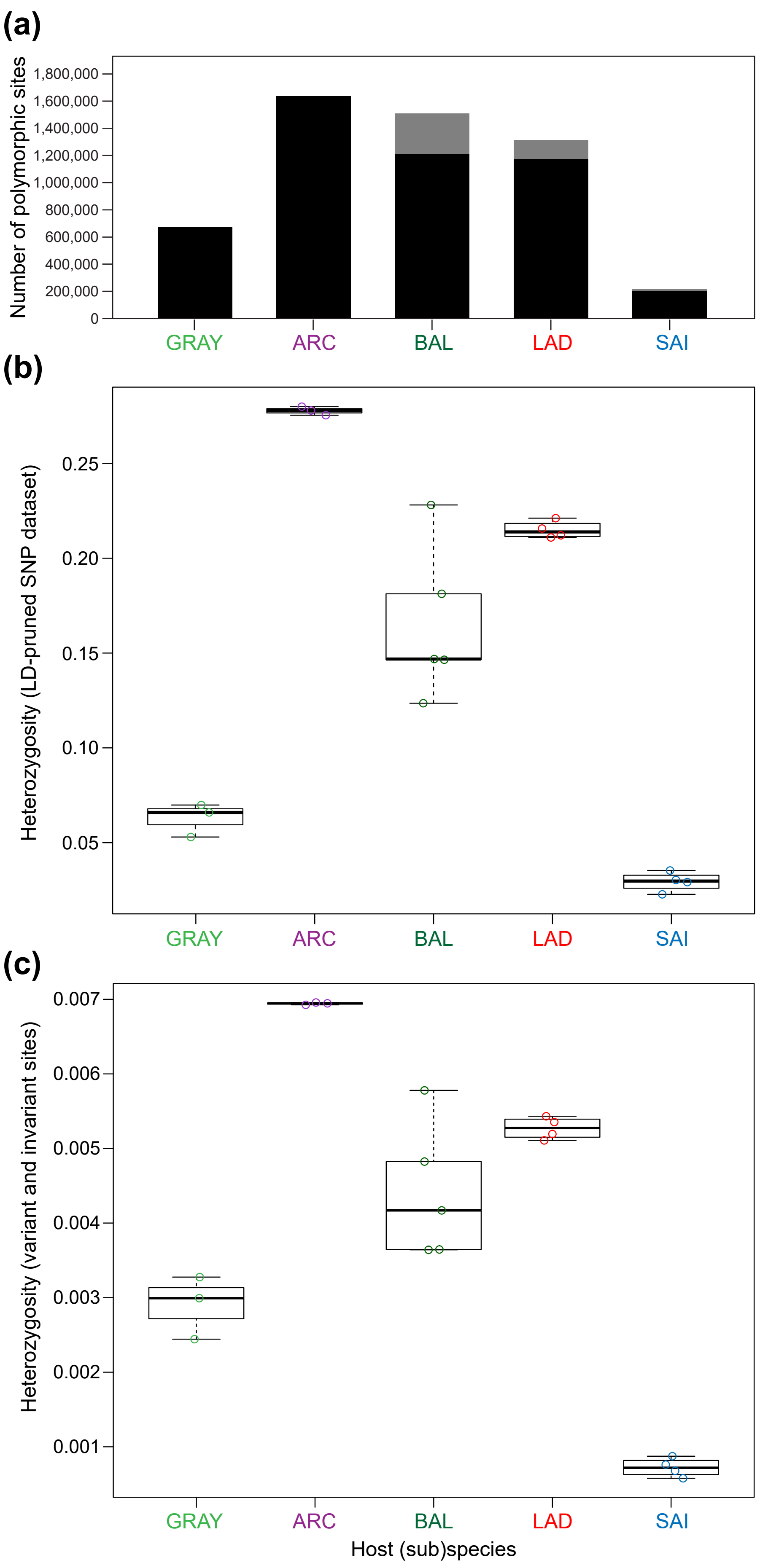
(a)
Number of polymorphic sites
1,800,000
1,600,000
1,400,000
1,200,000
1,000,000
800,000
600,000
400,000
200,000
0
GRAY
ARC
BAL
LAD
SAI
(b)
Heterozygosity (LD-pruned SNP dataset)
0.25
0.20
0.15
0.10
0.05
GRAY
ARC
BAL
LAD
SAI
(c)
Heterozygosity (variant and invariant sites)
0.007
0.006
0.005
0.004
0.003
0.002
0.001
GRAY
ARC
BAL
LAD
SAI
Host (sub)species

Fig. S4

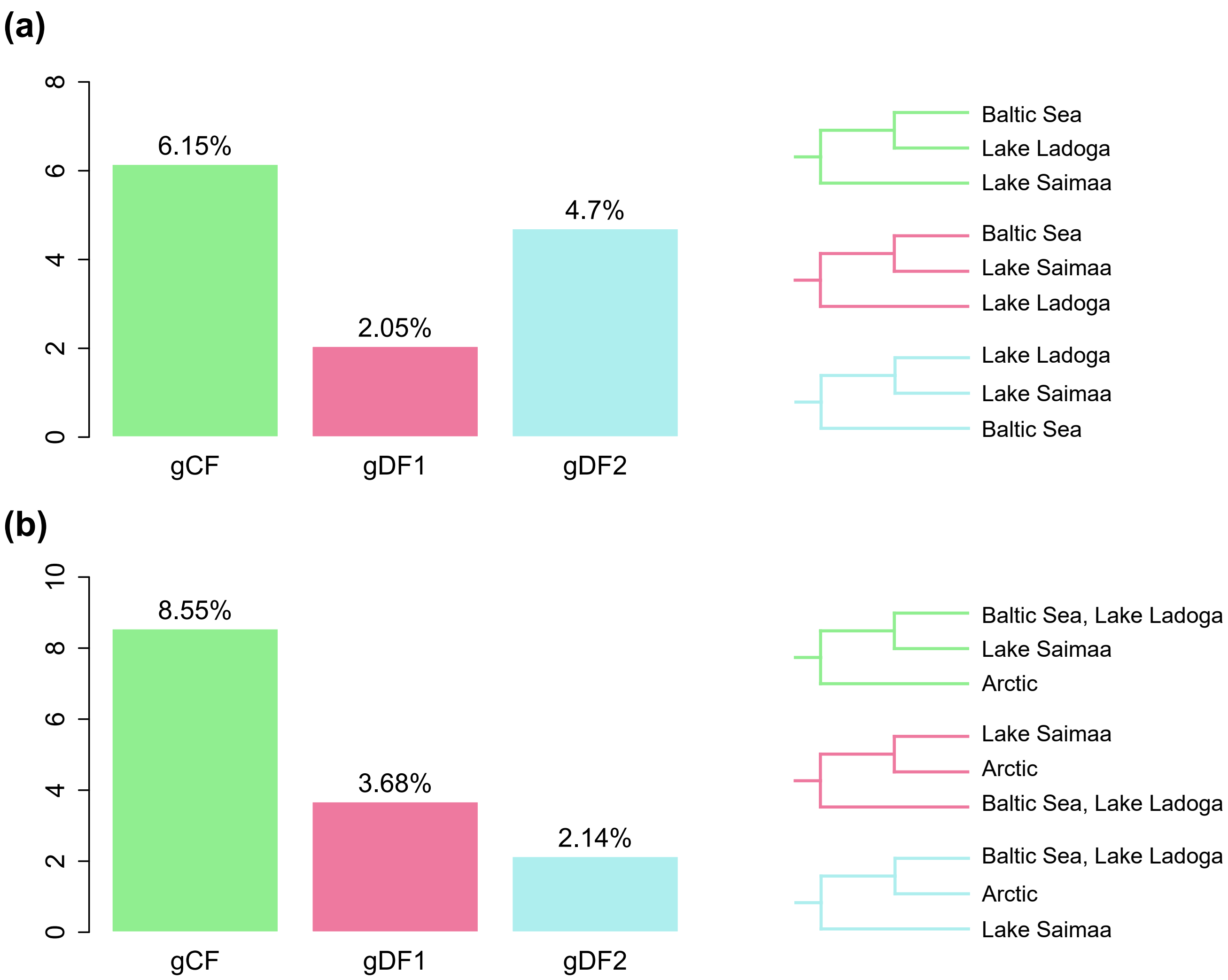
(a)
8
6
4
2
0
6.15%
2.05%
4.7%
gCF
gDF1
gDF2
Baltic Sea
Lake Ladoga
Lake Saimaa
Baltic Sea
Lake Saimaa
Lake Ladoga
Lake Ladoga
Lake Saimaa
Baltic Sea
(b)
10
8
6
4
2
0
8.55%
3.68%
2.14%
gCF
gDF1
gDF2
Baltic Sea, Lake Ladoga
Lake Saimaa
Arctic
Lake Saimaa
Arctic
Baltic Sea, Lake Ladoga
Baltic Sea, Lake Ladoga
Arctic
Lake Saimaa

Fig. S5

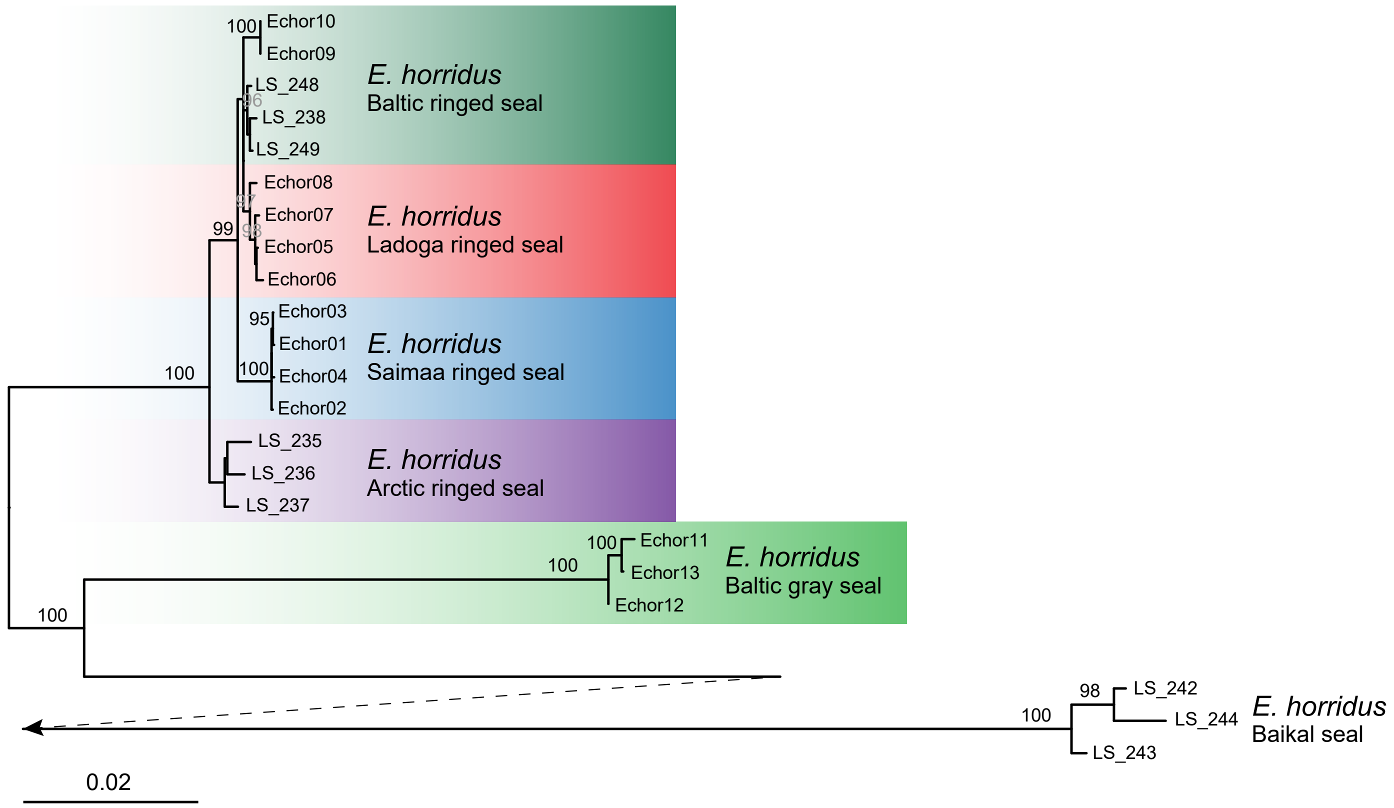
100
Echor10
Echor09
LS_248
96
LS_238
LS_249
E. horridus
Baltic ringed seal
Echor08
97
Echor07
99
98
Echor05
Echor06
E. horridus
Ladoga ringed seal
95
Echor03
Echor01
100
Echor04
Echor02
E. horridus
Saimaa ringed seal
100
LS_235
LS_236
LS_237
E. horridus
Arctic ringed seal
100
Echor11
100
Echor13
Echor12
E. horridus
Baltic gray seal
100
98
LS_242
LS_244
100
LS_243
E. horridus
Baikal seal
0.02

Fig. S6

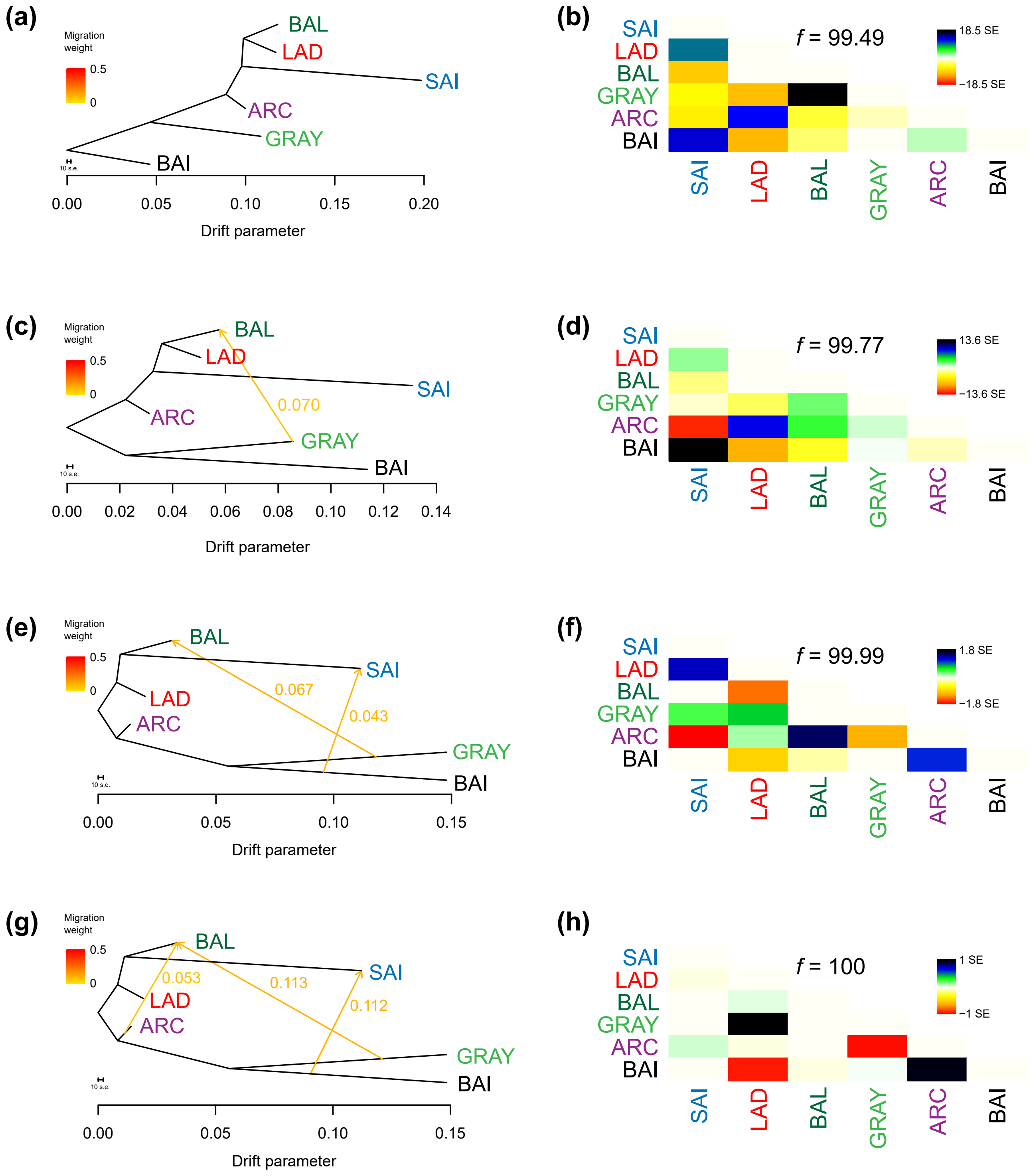

(a)
Migration weight
0.5
0
BAL
LAD
SAI
ARC
GRAY
BAI
10 s.e.
0.00
0.05
0.10
0.15
0.20
Drift parameter
(b)
SAI
LAD
BAL
GRAY
ARC
BAI
f = 99.49
18.5 SE
−18.5 SE
(c)
Migration weight
0.5
0
BAL
LAD
SAI
ARC
0.070
GRAY
BAI
10 s.e.
0.00
0.02
0.04
0.06
0.08
0.10
0.12
0.14
Drift parameter
(d)
f = 99.77
13.6 SE
−13.6 SE
(e)
Migration weight
0.5
0
BAL
SAI
0.067
0.043
LAD
ARC
GRAY
BAI
10 s.e.
0.00
0.05
0.10
0.15
Drift parameter
(f)
f = 99.99
1.8 SE
−1.8 SE
(g)
Migration weight
0.5
0
BAL
SAI
0.053
0.113
0.112
LAD
ARC
GRAY
BAI
10 s.e.
0.00
0.05
0.10
0.15
Drift parameter
(h)
f = 100
1 SE
−1 SE

Fig. S7

Table S1. Collection data and sequencing statistics for the 22 *Echinophthirius horridus* specimens analyzed in this study.

Louse individual code	Host (sub)species	Locality	Host individual code	Collection date	Sequencing centre[1]	Library construction kit	Number of reads	% duplicates[2]	Mean mapping depth
Echor01	*P. h. saimensis*	Pihlajavesi, Lake Saimaa	MI-13	3-Jun-2013	CBC	KAPA Hyper Prep KIt	63 426 734	14.1	45.88
Echor02	*P. h. saimensis*	Pihlajavesi, Lake Saimaa	TU-14	15-Apr-2014	CBC	KAPA Hyper Prep KIt	132 727 202	13.2	96.95
Echor03	*P. h. saimensis*	Haukivesi, Lake Saimaa	US-12	1-Jun-2012	CBC	KAPA Hyper Prep KIt	144 924 120	12.9	105.57
Echor04	*P. h. saimensis*	Haukivesi, Lake Saimaa	PA-09	22-May-2009	CBC	KAPA Hyper Prep KIt	125 049 252	15.4	87.07
Echor05	*P. h. ladogensis*	Valaam Archipelago, Lake Ladoga	Phl2-17	29-Jun-2017	CBC	KAPA Hyper Prep KIt	57 729 972	14.1	38.13
Echor06	*P. h. ladogensis*	Valaam Archipelago, Lake Ladoga	Phl-1-17	28-Jun-2017	CBC	KAPA Hyper Prep KIt	41 275 724	13.0	28.82
Echor07	*P. h. ladogensis*	Valaam Archipelago, Lake Ladoga	Phl2016-2	2016	CBC	KAPA Hyper Prep KIt	115 821 420	14.7	83.80
Echor08	*P. h. ladogensis*	Valaam Archipelago, Lake Ladoga	Phl2017-1	Aug-2017	CBC	KAPA Hyper Prep KIt	97 436 732	14.0	66.96
Echor09	*P. h. botnica*	Simo, Bothnian Bay, Baltic Sea	KU-13	06-Nov-2013	CBC	KAPA Hyper Prep KIt	68 838 182	17.3	45.12
Echor10	*P. h. botnica*	Simo, Bothnian Bay, Baltic Sea	KU-13	06-Nov-2013	CBC	KAPA Hyper Prep KIt	141 066 090	13.9	101.14
LS_238	*P. h. botnica*	Härkäletto, Bothnian Bay, Baltic Sea	Pup 1	16-Apr-2020	Genewiz	NEBNext Ultra II DNA Library Prep Kit	133 571 592	6.2	90.47
LS_248	*P. h. botnica*	Härkäletto, Bothnian Bay, Baltic Sea	Pup 1	16-Apr-2020	Genewiz	NEBNext Ultra II DNA Library Prep Kit	167 751 188	6.7	109.89
LS_249	*P. h. botnica*	Härkäletto, Bothnian Bay, Baltic Sea	Pup 2	16-Apr-2020	Genewiz	NEBNext Ultra II DNA Library Prep Kit	136 588 224	7.0	87.78
LS_235	*P. h. hispida*	Upernavik, Greenland	1	2020	Genewiz	NEBNext Ultra II DNA Library Prep Kit	260 799 826	9.2	127.48
LS_236	*P. h. hispida*	Upernavik, Greenland	1	2020	Genewiz	NEBNext Ultra II DNA Library Prep Kit	143 304 896	8.8	85.88
LS_237	*P. h. hispida*	Upernavik, Greenland	1	2020	Genewiz	NEBNext Ultra II DNA Library Prep Kit	257 130 462	8.2	140.13
Echor11	*H. grypus*	Kalmar County, Baltic Sea	A2016/05154	10-Mar-2016	CBC	KAPA Hyper Prep KIt	103 896 800	14.2	74.85
Echor12	*H. grypus*	Bothnian Sea, Baltic Sea	A2013/05611	20-Nov-2013	CBC	KAPA Hyper Prep KIt	74 073 304	14.2	52.85
Echor13	*H. grypus*	Stockholm Archipelago, Baltic Sea	A2012/05926	05-Dec-2012	CBC	KAPA Hyper Prep KIt	129 531 292	15.8	85.99
LS_242	*P. sibirica*	Lake Baikal	16	2017	Genewiz	NEBNext Ultra II DNA Library Prep Kit	173 702 278	34.8[3]	111.89
LS_243	*P. sibirica*	Lake Baikal	8	19-Apr-2017	Genewiz	NEBNext Ultra II DNA Library Prep Kit	153 993 034	45.2[3]	97.42
LS_244	*P. sibirica*	Lake Baikal	3	13-Apr-2017	Genewiz	NEBNext Ultra II DNA Library Prep Kit	218 932 232	9.3	128.16

[1] CBC: The Roy J. Carver Biotechnology Center, University of Illinois, United States; Genewiz: Genewiz from Azenta Life Sciences, Germany

[2] Estimated in BBTools.

[3] Calculated from combined data of two sequencing runs.

Table S2. Between- and within-group uncorrected percent COI sequence divergence among lice sampled from different seal hosts. Numbers represent means across all pairwise comparisons of sequences, with minimum and maximum values given in parentheses.

	**Saimaa ringed seal**	**Ladoga ringed seal**	**Baltic ringed seal**	**Arctic ringed seal**	**Baltic gray seal**	*N*	**Within group**
**Saimaa ringed seal**						4	0.04% (0–0.07)
**Ladoga ringed seal**	0.37% (0.35–0.42)					4	0% (0–0)
**Baltic ringed seal**	0.42% (0.35–0.56)	0.20% (0.14–0.28)				5	0.18% (0–0.28)
**Arctic ringed seal**	0.94% (0.84–1.05)	0.58% (0.49–0.70)	0.72% (0.49–0.98)			3	0.19% (0.07–0.28)
**Baltic gray seal**	5.61% (5.53–5.67)	5.42% (5.39–5.46)	5.56% (5.46–5.67)	5.35% (5.25–5.46)		3	0.09% (0.07–0.14)
**Baikal seal**	12.68% (12.61–12.75)	12.61% (12.54–12.68)	12.55% (12.39–12.75)	12.89% (12.75–13.03)	12.70% (12.54–12.89)	3	0.51% (0.35–0.63)

N – number of individuals

Table S3. Concordance factor statistics
ID: Branch ID (see tree on the right)
gCF: Gene concordance factor (=gCF_N/gN %)
gCF_N: Number of trees concordant with the branch
gDF1: Gene discordance factor for NNI-1 branch (=gDF1_N/gN %)
gDF1_N: Number of trees concordant with NNI-1 branch
gDF2: Gene discordance factor for NNI-2 branch (=gDF2_N/gN %)
gDF2_N: Number of trees concordant with NNI-2 branch
gDFP: Gene discordance factor due to polyphyly (=gDFP_N/gN %)
gDFP_N: Number of trees decisive but discordant due to polyphyly
gN: Number of trees decisive for the branch
sCF: Site concordance factor averaged over 100 quartets (=sCF_N/sN %)
sCF_N: sCF in absolute number of sites
sDF1: Site discordance factor for alternative quartet 1 (=sDF1_N/sN %)
sDF1_N: sDF1 in absolute number of sites
sDF2: Site discordance factor for alternative quartet 2 (=sDF2_N/sN %)
sDF2_N: sDF2 in absolute number of sites
sN: Number of informative sites averaged over 100 quartets
Label: Existing branch label (i.e. bootstraps values)
Length: Branch length
NOTE: (gCF+gDF1+gDF2+gDFP) = 100% and (gCF_N+gDF1_N+gDF2_N+gDFP_N) = gN

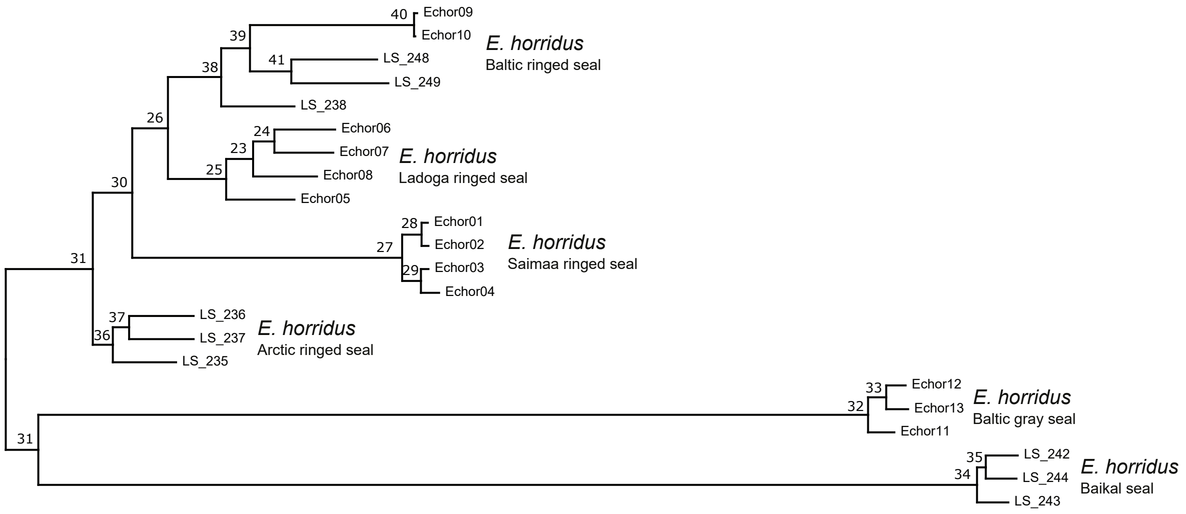

ID	gCF	gCF_N	gDF1	gDF1_N	gDF2	gDF2_N	gDFP	gDFP_N	gN	sCF	sCF_N	sDF1	sDF1_N	sDF2	sDF2_N	sN	Label	Length
23	10,68	125	7,35	86	12,22	143	69,74	816	1170	37,91	3940,47	32,15	3340,55	29,94	3113,91	10394,93	100	0,000256
24	16,32	191	14,7	172	17,61	206	51,37	601	1170	36,96	3560,09	28,21	2716,63	34,83	3367,1	9643,82	98	0,000204
25	11,97	140	1,62	19	1,37	16	85,04	995	1170	56,71	10671,41	22,13	4112,97	21,16	3954,43	18738,81	100	0,000562
26	6,15	72	2,05	24	4,7	55	87,09	1019	1170	37,84	13091,02	28,35	9581,87	33,81	11671,49	34344,38	100	0,000337
27	87,26	1021	0,09	1	0,09	1	12,56	147	1170	96,32	68120,3	1,71	1228,11	1,97	1422,02	70770,43	100	0,002585
28	34,02	398	17,09	200	17,09	200	31,79	372	1170	75,34	3977,4	15,8	817,49	8,85	507,66	5302,55	100	0,000187
29	32,65	382	23,08	270	17,01	199	27,26	319	1170	59,2	4123,55	29,07	2035,09	11,73	815,59	6974,23	100	0,00018
30	8,55	100	3,68	43	2,14	25	85,64	1002	1170	40,84	14019,39	31,88	10946,37	27,29	9462,67	34428,43	100	0,000378
31	69,74	816	0,17	2	0,6	7	29,49	345	1170	73,49	43631,97	12,85	7804,74	13,66	8225,14	59661,85	100	0,00115
32	95,81	1121	0	0	0,94	11	3,25	38	1170	98,82	236774,3	0,48	1153,3	0,7	1675,98	239603,6	100	0,007952
33	33,08	387	31,37	367	35,3	413	0,26	3	1170	53,98	4659,31	23,01	1985,94	23,01	1985,13	8630,38	100	0,000173
34	100	1170	0	0	0	0	0	0	1170	99,83	268062,3	0,09	234,73	0,08	217,07	268514,1	100	0,008996
35	36,24	424	32,91	385	30,85	361	0	0	1170	35,15	2257,98	32,41	2081,6	32,44	2083,54	6423,12	100	7,96E-05
36	9,57	112	6,5	76	3,85	45	80,09	937	1170	46,42	8725,23	26,16	4832,22	27,43	5080,47	18637,92	100	0,00019
37	13,93	163	15,21	178	14,27	167	56,58	662	1170	34,07	3076,66	33,39	3009,07	32,55	2941,82	9027,55	93	0,000159
38	13,93	163	2,74	32	0,94	11	82,39	964	1170	44,39	8734,72	29,84	5964,57	25,77	5128,33	19827,62	100	0,00051
39	6,84	80	12,82	150	7,26	85	73,08	855	1170	33,4	6245,69	31,2	5913,86	35,4	6637,04	18796,59	100	0,000278
40	72,65	850	0,77	9	0,43	5	26,15	306	1170	99,27	31506,08	0,21	65,7	0,52	168,33	31740,11	100	0,00157
41	27,78	325	6,84	80	16,07	188	49,32	577	1170	43,27	9086,68	22,58	4813,09	34,15	7143,3	21043,07	100	0,000397
